# Scalable intermediate-term earthquake forecasting with multimodal fusion neural networks

**DOI:** 10.1038/s41598-025-93877-7

**Published:** 2025-03-21

**Authors:** Yumeng Hu, Qi Zhang, Hengshu Zhu, Baoshan Wang, Hui Xiong, Haitao Wang

**Affiliations:** 1 School of Earth and Space Sciences, University of Science and Technology of China, Hefei, 230026 China; 2 Shanghai Artificial Intelligence Laboratory, Shanghai, 200232 China; 3 Computer Network Information Center, Chinese Academy of Sciences, Beijing, 100083 China; 4 Thrust of Artificial Intelligence, The Hong Kong University of Science and Technology (Guangzhou), Guangzhou, 511458 China; 5 China Earthquake Networks Center, China Earthquake Administration, Beijing, 100045 China; 6 Department of Computer Science and Engineering, The Hong Kong University of Science and Technology, Hong Kong SAR, China

**Keywords:** Natural hazards, Seismology, Computational science, Computer science

## Abstract

Seismology is witnessing rapid growth in both the volume and variety of earthquake observational data, but current tools for effectively integrating these heterogeneous data remain limited. Here, we propose SafeNet, a scalable deep learning framework designed to address these challenges through the use of multimodal fusion neural networks. SafeNet integrates 282-dimensional seismic indicators from earthquake catalogs, capturing long-, medium-, and short-term seismic patterns, and associates seismic activity with geological information using integrated maps. Its specialized fusion modules and adaptive attention mechanism enable dynamic spatiotemporal information exchange across regions. To validate SafeNet’s performance, we conducted a pseudo-prospective test using a 50-year earthquake catalog from China, demonstrating its superior forecasting performance over 13 state-of-the-art models. Additionally, the successful transfer of models trained on the China dataset to the Contiguous and Western United States further highlights SafeNet’s scalability.

## Introduction

As a major natural geological disaster, earthquakes cause significant economic and human losses worldwide each year^1,2^. Public demand for reports detailing the severity, probability, and location of future earthquake hazards has intensified, favoring layered maps-based forecasts for clearer visualization^3^. To address this, researchers and governments have expanded seismic networks and adopted automated data processing, seeking to improve the accuracy of forecasts. As earthquake catalogs and observational data grow, new forecasting methods are being developed^4–7^. However, forecasting intermediate-term $M\ge 5$ earthquakes across large-scale space, diverse tectonic environments remains a challenge^8,9^. One possible explanation lies in the theoretical limitations of existing models^10^. Many of these models are rooted in classical seismic theories designed for sparse datasets^11–13^, making it difficult to efficiently leverage the dense and diverse observational data^14–16^. Additionally, the limited historical samples of $M \ge 5$ earthquakes in each region hinder these models from effectively capturing their statistical patterns.

Machine learning (ML) emerges as a promising solution to these challenges^8,17^. Its potential, already evident in laboratory settings, lies in its ability to identify patterns that traditional methods overlook^18–20^, has now expanded to natural earthquake forecasting^8,17^. Recent research has applied ML models to seismic indicators like the b-value from earthquake catalogs, validating their use as time series inputs and emphasizing the importance of temporal patterns^21,22^. In parallel, using convolutional neural networks (CNNs) to analyze earthquake distribution maps highlights the critical role of spatial relationships in earthquake forecasting^23–25^. While these approaches provide enhanced computational efficiency and greater resilience to nonstationarity in earthquake catalogs compared to classical models like the Epidemic-Type Aftershock Sequence (ETAS)^14,26–28^, they still struggle with capturing complex spatio-temporal dynamics and integrating heterogeneous data. Existing ML models for earthquake forecasting are often trained and tested within the same geographic region, limiting the ability to assess their generalizability across diverse seismic environments^23–25^. This regional focus creates a challenge in comparing model performance across different seismic backgrounds. Moreover, inconsistent evaluation metrics and the lack of standardized baselines further complicate comparisons, hindering progress in the field of earthquake forecasting based on ML. For instance, in some cases, simpler, shallow networks may exhibit performance similar to more complex, deep models, which can obscure the true potential of advanced techniques and stall model advancement^29,30^. The absence of transparent, consistent evaluation practices, coupled with potential biases in regional datasets, underscores the need for a more rigorous approach to model assessment^31^.

In this study, we introduce SafeNet, a powerful multimodal deep learning-based earthquake forecasting model that can swiftly generate predictions for earthquake magnitudes in a specific region for the upcoming year within seconds. We evaluated SafeNet against 13 benchmark models using standard machine learning classification metrics on a dataset covering 85 regions in China, facilitating comparison and future improvements. SafeNet outperformed all benchmark models in this comparison. Additionally, we demonstrated its transferability and scalability by successfully applying it to datasets from the contiguous U.S. and western U.S.

The design of SafeNet offers two key contributions: First, SafeNet enables the integration of diverse data sources into forecasting processes. This multimodal fusion offers a comprehensive spatial-temporal perspective, and its scalable design establishes a foundation for further exploration of the contributions from various geophysical datasets. Another key feature of our model is its ability to exchange information across all regions. This not only mitigates the challenges posed by the scarcity of high-magnitude earthquake events, but also enhances forecasting through learned tectonic context. Consequently, SafeNet emerges as an efficient and scalable tool for giving layered forecast maps for diverse geologic environments.

## Results

**Figure 1:**
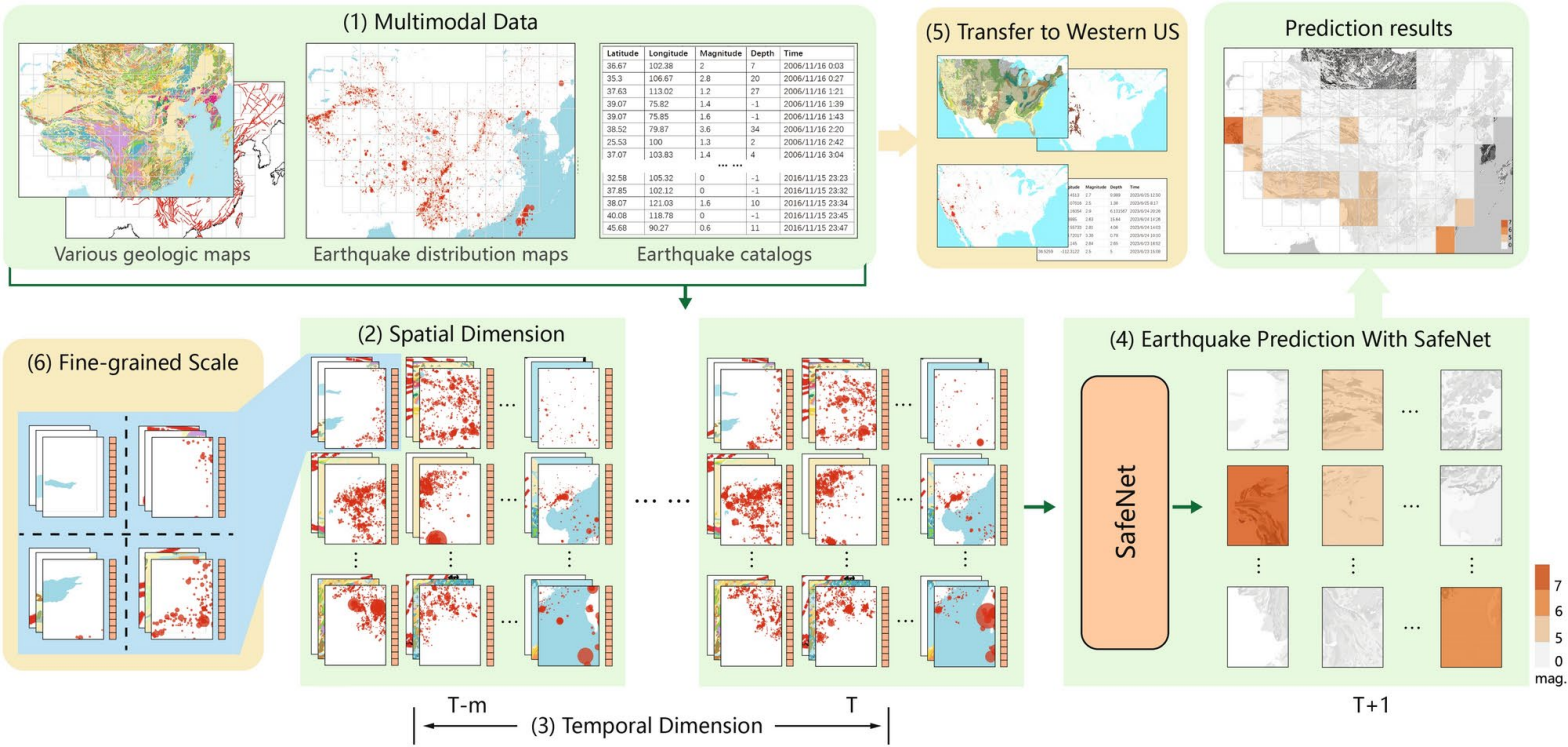
A schematic diagram of SafeNet for earthquake forecasting. (1) Various geologic and seismic maps are modeled as images and fused with earthquake catalogs for earthquake prediction. Maps were plotted using the Generic Mapping Tools Version 6 (GMT6)^32^. See Methods for details. (2) The image of each map is divided into many small regions (i.e., image patches) to extract spatial features. (3) The region-based features of images in different time periods are formed as a multi-source, multimodal time series. (4) With a specially-designed Vision Transformer network, the multimodal time series features from all regions are utilized for predicting future earthquake occurrences in each region. (5) Transfer SafeNet to a new region, fine-tuning it with multimodal data from the United States. (6) Divide the regions into more fine-grained scale, allowing SafeNet to predict earthquake in the new scale grid.

**Figure 2:**
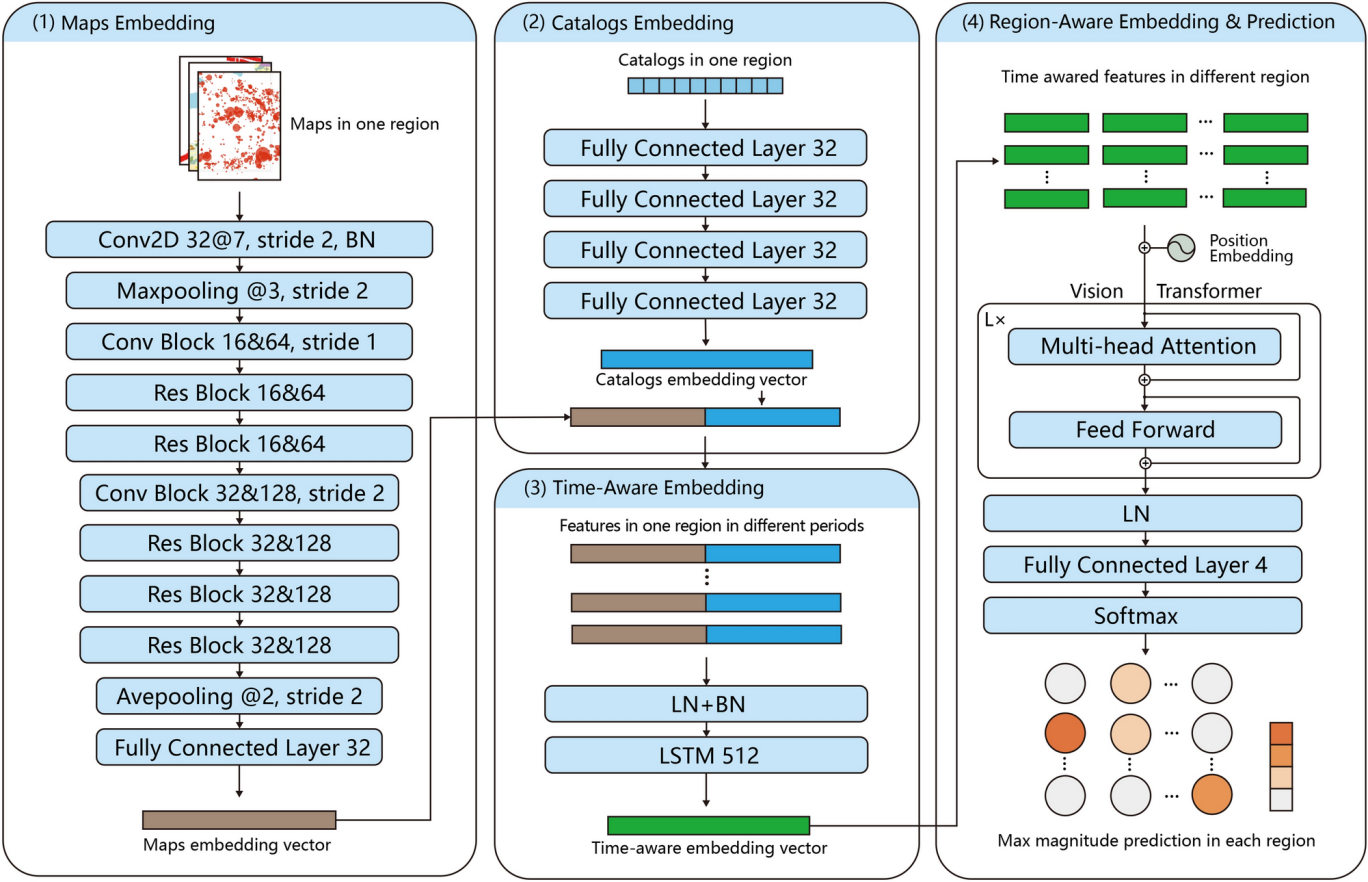
The network structure of SafeNet. (1) Maps Embedding: Extracts embeddings from image patch data of various maps for each region. Maps were plotted using GMT 6^32^. (2) Catalogs Embedding: Extracts the embedding of earthquake catalog-based features in each region, combined with maps embeddings for comprehensive regional earthquake information. (3) Time-Aware Embedding: Models the temporal patterns of earthquakes in each region, generating time-aware patch embeddings of multimodal features. (4) Region-Aware Embedding & Prediction: Constructs relationships and information communications among different regional patch embeddings using a Vision Transformer, predicting the maximum magnitude range of earthquakes in each region.

**Table 1:** Details of catalog indicators.

Feature	Dim	Description
Top 5 highest earthquakes’ magnitude	65	Retrieves the top 5 earthquake magnitudes for the previous year and for each month within that year
Lunar date	65	Retrieves the lunar dates of the top 5 highest earthquake magnitudes for the previous year and for each month within that year, and use $f(x)=sin(x/N*2\pi )+2$ to normalize, which *x* represents the day and *N* represents the total days in its month (i.e. 29 days or 30 days)
Different magnitude event count	48	Counts seismic events for each month of the previous year in four distinct magnitude ranges: $0\le M \le 3$, $3\le M \le 5$, $5\le M \le 7$ and $7\le M$
Different depth event count	24	Counts the number of seismic events with depths greater than and less than 70km for each month of the previous year
b value	14	Extract the b value according to the G-R relation for the previous year, each month of that year, and the latest 100 events
a value	14	Extract the a value according to the G-R relation for the previous year, each month of that year, and the latest 100 events
Mean magnitude	14	Calculates the mean of magnitude for the previous year, each month of that year, and the latest 100 events
Standard deviation	13	Calculates the standard deviation of magnitude for the previous year and for each month within that year
Probability dense of $M\ge 6$	13	Calculates the probability dense of $M\ge 6$ for the previous year and for each month within that year^33^
a/b	1	Calculates the a value divided by the b value for the latest 100 events
MSE of G-R model	1	Calculates the mean square e estimation for the G-R model for the latest 100 events^21^
$\Delta T$	1	Computes the time difference between the 100th event and the 1st event ($T_{100} -T_1$)^21^
*dE*	1	Calculates the root mean square energy of the latest 100 events^21^
Mean of $\Delta T_{n+1} -T_n$	4	Calculates the interval time for four magnitude range: $0\le M < 3$, $3\le M < 5$, $5\le M < 7$ and $7\le M$
Std/Mean of $\Delta T_{n+1} -T_n$	4	Calculates the ratio of standard deviation time difference to mean time difference for each magnitude range, specifically for $0\le M < 3$, $3\le M < 5$, $5\le M < 7$ and $7\le M$

### Framework overview

Figure 1 illustrates the SafeNet model, which predicts earthquake magnitudes by fusing multimodal geologic and seismic data to analyze spatio-temporal correlations in diverse seismic regions. To analyze spatio-temporal correlations in diverse seismic regions, SafeNet uniquely integrates multimodal features across a range of time scales, from long-term historical data to annual, monthly, and daily seismic trends. As catalog input, a total of 282 catalog indicators are analyzed (Table 1), including time-based indicators (e.g., intervals between earthquakes and lunar dates), location-based indicators (e.g., depth, latitude, and longitude), and magnitude-based indicators (e.g., earthquake magnitude and the Gutenberg-Richter relationship). The details can be found in Methods. This integration is crucial for capturing a comprehensive seismic background in each region and addressing the challenge of sparse strong earthquake data. Table S1 provides further evidence that expanding the analysis to include 282 indicators substantially enhances prediction accuracy. To fully capture the spatio-temporal features of seismicity, we designed four main modules (detailed in Fig. 2), with their efficacy validated through ablation experiments presented in Table S2. The *Maps Embedding module* processes diverse maps into fine-grained regions, employing a ResNet-50^34^ architecture to extract and embed geologic and seismicity patterns into a multimodal space. The *Catalog Embedding module* further enriches this space with 282-dimensional features from earthquake catalogs per region, integrated through four fully-connected layers. To capture temporal earthquake patterns, the *Time-Aware Embedding module* transforms these comprehensive embeddings into time series, using a Long Short-Term Memory (LSTM) network^35,36^. As the larger the impending earthquake, the longer the period and wider the area covered by its precursors. Considering this, the final module, *Region-Aware Embedding & Prediction*, leverages a Vision Transformer Encoder^37^ for understanding inter-regional relationships with an attention mechanism^38^. This communication enables the model to dynamically adjust its focus, giving more weight to the integrated features from regions that are most relevant to the prediction of seismic activity in a target region. This comprehensive approach, culminating in a linear layer, is designed to predict the maximum earthquake magnitude for each region in the upcoming year.

### Data preprocessing

To validate the feasibility of SafeNet, we conducted extensive experiments on a real-world dataset provided by the China Earthquake Networks Center (CENC), which includes the earthquake catalogs and corresponding geologic maps. This catalog data spans nearly 52 years, covering earthquake magnitude $M _L-1.9$ $\sim$ 8.1 across most of China, and contains 1,509,163 earthquake records from January 1, 1970, to November 16, 2021. The earthquake distribution is extremely imbalanced both in magnitude and space (Figs. S1 and S2). The research area extended from latitude $20^{\circ }$ to $50^{\circ }$ and longitude $73^{\circ }$ to $133^{\circ }$, divided into 120 regions by a $4^{\circ }\times 4^{\circ }$ grid. We then deleted the regions with no data, resulting in 85 research regions. Additionally, we considered the entire area as another new region (the global token region No.0) for feature extraction to help the model generate a macroscopic seismic activity background. To enhance our training dataset, we extracted features for each day (*t*) that summarized the seismic activity of the previous 365 days, expanding the sample size from 51 annual to 18,584 daily records. Subsequently, we incorporated spatial information by integrating a Generalized Geology Map, a detailed map of China’s primary fault, and the corresponding annual earthquake distribution map with the day’s seismic catalog feature to form a multimodal feature. To capture both temporal and spatial seismic variations efficiently, we assembled ten sets ($T=10$) of these multimodal features to constitute a comprehensive multimodal input. Time-window length experiments for *T* are detailed in Table S3. For labeling, we used a four-class system based on the magnitude $M$ of the largest earthquake in each region for the year following time $t$: $0 \le M < 5$, $5 \le M < 6$, $6 \le M < 7$, and $7 \le M$. The training dataset consists of multimodal inputs from January 1, 1979, to November 15, 2006. The validation dataset spans from November 16, 2005, to November 15, 2011, with labels containing information until November 15, 2012. Accordingly, the testing set covers November 16, 2012, to November 16, 2020, with labels extending up to our last available data on November 16, 2021.

### Earthquake prediction results across 85 regions in China

**Table 2:** Overall performance of different methods for earthquake prediction.

Model	Accuracy$$^{*}$$	Macro F1$$^{**}$$	$$0 \le M < 5$$			$$5 \le M < 6$$			$$6 \le M < 7$$			$$7 \le M$$		
			F1	Recall	Precision	F1	Recall	Precision	F1	Recall	Precision	F1	Recall	Precision
GBDT	0.7542	0.2572	0.8684	**0.9809**	0.7790	0.1605	0.0922	**0.6190**	0.0000	0.0000	0.0000	0.0000	0.0000	0.0000
LR	0.7490	0.2655	0.8609	0.9635	0.7781	0.2011	0.1348	0.3958	0.0000	0.0000	0.0000	0.0000	0.0000	0.0000
RNN	0.7412	0.2786	0.8567	0.9357	0.7900	0.2578	0.2057	0.3452	0.0000	0.0000	0.0000	0.0000	0.0000	0.0000
SVM	0.7673	0.2875	0.8689	0.9739	0.7843	0.2812	0.1915	0.5294	0.0000	0.0000	0.0000	0.0000	0.0000	0.0000
LSTM	0.7451	0.3030	0.8574	0.9357	0.7912	0.2830	0.2128	0.4225	0.0714	0.0476	0.1429	0.0000	0.0000	0.0000
RF	0.7686	0.3063	0.8703	0.9565	0.7983	0.3551	0.2695	0.5205	0.0000	0.0000	0.0000	0.0000	0.0000	0.0000
DNN	0.6967	0.3124	0.8221	0.8557	0.7910	0.2941	0.2837	0.3053	0.0000	0.0000	0.0000	0.1333	0.1429	0.1250
CNNbilstmA	0.6484	0.3149	0.7815	0.7496	0.8163	0.3446	**0.4326**	0.2864	0.1333	0.0952	0.2222	0.0000	0.0000	0.0000
PRNN	0.6431	0.3150	0.7859	0.7565	0.8177	0.2967	0.3546	0.2551	0.1772	0.1667	0.1892	0.0000	0.0000	0.0000
Boosting	0.7608	0.3225	0.8720	0.9304	0.8206	0.3755	0.3262	0.4423	0.0426	0.0238	0.2000	0.0000	0.0000	0.0000
Transformer	0.6902	0.3438	0.8206	0.8435	0.7990	0.2909	0.2837	0.2985	0.0635	0.0476	0.0952	0.2000	0.1429	0.3333
**SafeNet**	**0.7699**	**0.4421**	**0.8748**	0.9113	**0.8411**	**0.4302**	0.4043	0.4597	**0.2414**	**0.1667**	**0.4375**	**0.2222**	**0.1429**	**0.5000**

Model	Accuracy$$^{***}$$	Runtime of Predictions				$$5 \le M$$			$$6 \le M$$			$$7 \le M$$		
						F1	Recall	Precision	F1	Recall	Precision	F1	Recall	Precision
ETAS	0.8029	Over 270 h				0.5313	0.4497	**0.6489**	0.0741	0.0408	0.4000	0.0000	0.0000	0.0000
Fast-ETAS	0.7858	Over 110 h				0.4792	0.3968	0.6048	0.0317	0.0204	0.0714	0.0000	0.0000	0.0000
**SafeNet**	**0.8042**	**Under 1 min**				**0.5498**	**0.4989**	0.6410	**0.2388**	**0.1633**	**0.4444**	**0.2222**	**0.1429**	**0.5000**

Accuracy*: Mainly reflects performance for $0 \le M < 5$, due to its higher frequency in the dataset. Utilizes a four-category classification.

Macro F1**: Mainly reflects performance for $M \ge 5$, calculated by averaging F1 scores across all four categories.

Accuracy***: Mainly represents performance for $M < 5$, due to its higher frequency. Uses a binary classification: $M < 5$, $M \ge 5$.

The upper section presents a comparative study of SafeNet against 11 leading machine learning baselines, showcasing the advancements across all earthquake magnitude range predictions. The lower section compares SafeNet with two advanced spatio-temporal ETAS models, using appropriate evaluation metrics and regions for ETAS. Bold numbers signify the best performance in each column.

After training SafeNet, we evaluated its performance on the test dataset by first generating the probability for each earthquake magnitude range, and then selecting the category with the highest probability as the prediction. The annual forecasting maps and the changes in probability are presented in Figs. S3–S7. Table 2 & Table S1 demonstrate SafeNet’s performance in earthquake forecasting, surpassing 5 classic machine learning models, 6 state-of-the-art deep learning models, and 2 advanced spatio-temporal ETAS methods^39^ in most evaluation metrics. For the 11 evaluated machine learning models (5 classical and 6 deep learning), SafeNet demonstrated superior performance in macro F1 scores, a key metric for balanced assessment on imbalanced datasets. This underscores SafeNet’s consistent effectiveness across all earthquake magnitude ranges. Although the ETAS model is optimal for describing the short-term evolution of aftershock sequences, which are typically marked by decreasing magnitudes rather than changes in background mainshock rates, it has shown some potential for long-term forecasting of large earthquakes^40^. In our tests, ETAS recalled 2 out of the 5 regions where it predicted $M\ge 6$ earthquakes, and its faster variant, Fast-ETAS, correctly identified 1 out of 14 regions. In comparison, SafeNet recalled 8 out of the 18 regions where it predicted $M\ge 6$ earthquakes, achieving this with a 15,000-fold increase in run time. These results and module ablation experiments (Table S2) verify the effectiveness of SafeNet, and make it a potential tool for big earthquakes forecasting in large scale. Furthermore, the paired *t*-tests and Wilcoxon tests results, as shown in Table S4, reveal that our model consistently provides a significant predictive advantage across 57 regions have previously experienced $M \ge 5$ earthquakes for each baseline method.

**Figure 3:**
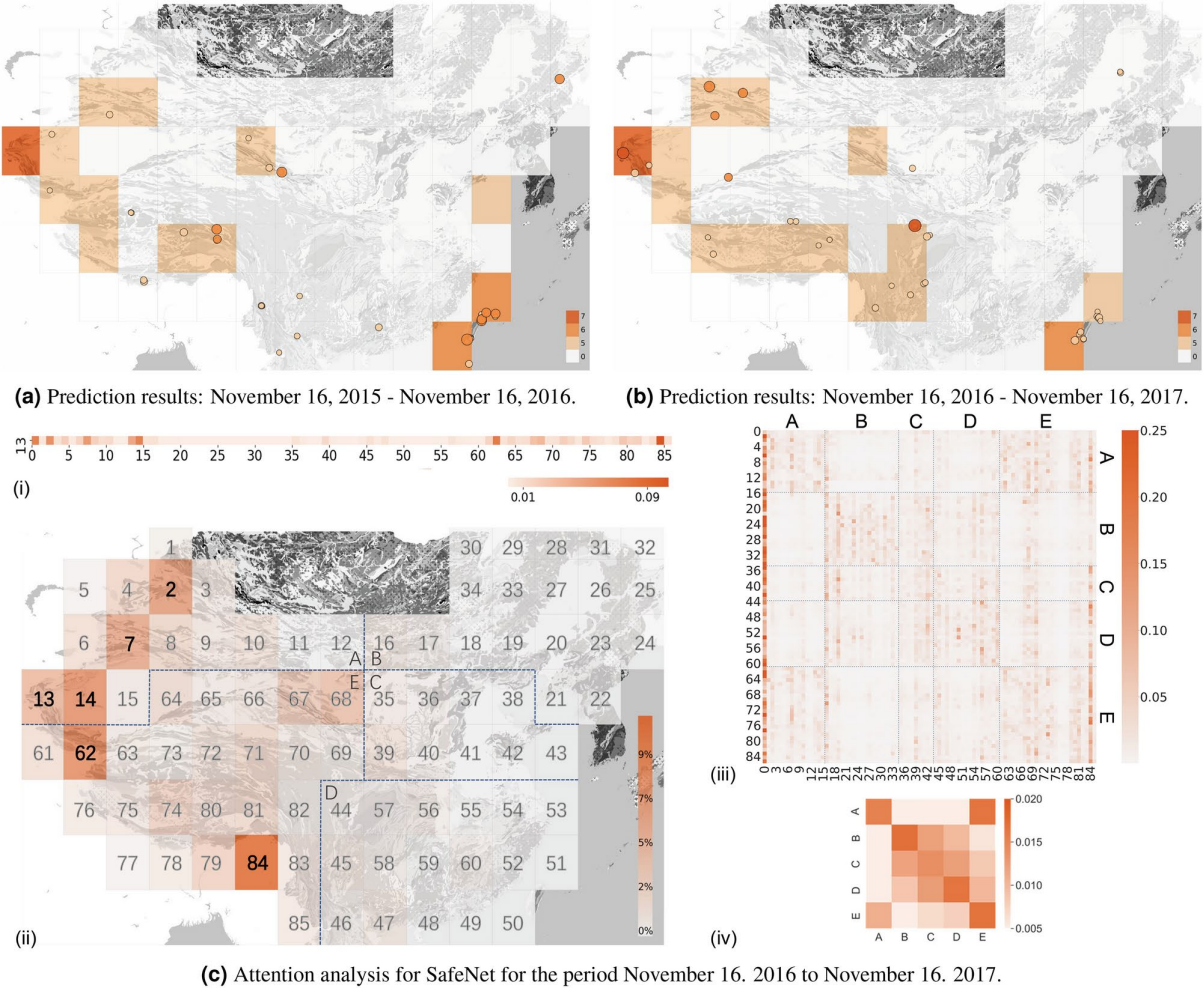
Case studies of prediction results. (**a**,**b**) The regions marked with color indicate where SafeNet predicts earthquakes of different ranges of magnitudes will occur, and the dots with color represent the ground truth of earthquakes with different magnitudes. **c**: interpreting the prediction mechanism, which demonstrates SafeNet’s ability to integrate localized seismic features with active tectonic relationships. **c**(i): The attention distribution of region No. 13 over all 85 regions (including itself) and the global region (No. 0 region). The depth of color denotes the impact on region No. 13’s earthquake prediction, with darker shades indicating greater influence. **c**(ii): The map view of **c**(i), highlighting that the Karakorum faults, Tien Shan system, Chinese Altai, and the regions affected by the northeastward movement of the Indian plate are important for the prediction of region No. 13. **c**(iii): The attention map of all regions, where each row represents the attention distribution of the current region to all 85 regions (including itself) and the global region (No. 0). **c**(iv): The attention map of all blocks, denoting SafeNet’s ability to build a wide-scale seismicity relationship. Maps were plotted using GMT 6^32^. See Methods for details.

To further assess the effectiveness of our model, we compared its predictions with the actual distribution of earthquakes with a magnitude of $M \ge 5$ (Fig. 3a,b, and Figs. S3–S7). SafeNet demonstrates potential in capturing key seismogenic patterns associated with strong earthquakes. Between 2015 and 2017, it predicted 10 of 13 ground truth earthquakes with magnitudes $6 \le M < 7$ in at-risk regions, accurately forecasting five of their magnitude ranges. For $M \ge 7$ earthquakes, the model correctly predicted one out of two, compared to seven actual occurrences in the test dataset. While accuracy for high-magnitude events is limited, its false positive prediction for an $M \ge 7$ region in 2015 was validated by a 2016 event, suggesting the model can anticipate significant seismic activity, albeit with some time error. Additionally, SafeNet’s ability to track seismic trends was evident in the southeast regions, where it successfully predicted a decline in earthquake magnitudes from 2015 to 2016, reflecting a decrease in actual seismic activity. These instances highlight SafeNet’s ability to adapt its predictions to dynamic seismic patterns. SafeNet’s adaptability is further evidenced by the changes in probability distributions (Figs. S3–S7), highlighting the model’s responsiveness to spatio-temporal changes. By integrating these prediction results with probability distributions, SafeNet provides a more comprehensive view of earthquake forecasting.

### Visualization of inter-regional relationships

SafeNet leverages an attention mechanism to model relationships between various regions, yielding detailed interpretations of earthquake predictions (Fig. 3c and Fig. S8). Regions are numbered based on fault distribution and active tectonic blocks^41–44^ and categorized into blocks A to E for easier visualization and analysis (details in Methods). First, we focus on region No. 13, successfully predicted to experience a $M\ge 7$ earthquake in 2016, and assess its influences from 85 regions, including a global token (region No. 0). Figure 3c(i) shows that predictions for region No. 13 are closely associated with multiple other regions. The locations of these regions are illustrated in Fig. 3c(ii) , which reveals a sophisticated recognition of seismic influences that extends beyond mere proximity. The map points seismic contributors like region No. 62, marking the end of the Karakorum Fault, and region No. 84, at the Indian-Eurasian plate boundary. Further, the high attention assigned to the Chinese Altai region (No. 2) resonates with its known function in accommodating compression stress. These details prove SafeNet’s ability to extract and utilize features from multiple related regions for each region’s prediction.

We further explore the attention map for prediction of all 85 regions (Fig. 3c(iii)). The map underscores the importance of a global seismic context (region No. 0), with a diagonal emphasis indicative of a focus on local and neighboring regions. Vertical alignments in key columns, such as No. 16 and No. 84, highlight the model’s attention to tectonic boundary areas. The interactions within and between the tectonic blocks A to E show the model’s sensitivity to tectonic dynamics, capturing first-order tectonic block characteristics (Fig. 3c(iv)). These patterns, particularly the connections between blocks A and E and the interactions among blocks B, C, and D, align with the impacts of known plate collision and subduction zones. This shows SafeNet’s ability to effectively combine localized seismic features with larger tectonic processes, providing a more comprehensive understanding of the seismogenic process across different scales.

**Figure 4:**
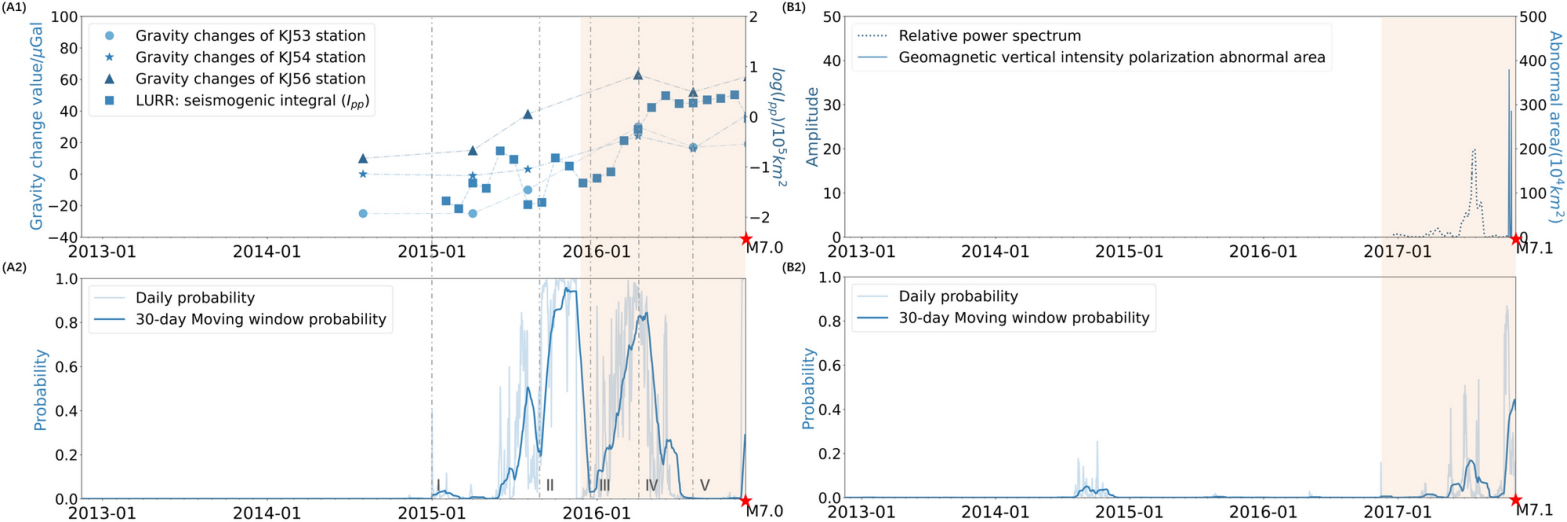
The comparisons between anomalies found in previous studies and the prediction probability change of SafeNet in regions of (**A**) *M*7.0 Aketao earthquakes and (**B**) *M*7.1 Milin earthquake. (**A1**) The gravity changes at three epicenter stations and the seismogenic integral curve on a logarithmic scale. (**A2**) SafeNet’s $M\ge 7$ probability trends, averaged using a 30-day moving window. (**B1**) The thermal infrared changes and the geomagnetic vertical strength polarization changes. (**B2**) SafeNet’s $M\ge 7$ probability trends, averaged using a 30-day moving window. The orange-shaded area represents the 1-year time window before the event.

### Change in $M \ge 7$ probability consistent with observed anomalies

SafeNet shows probability shifts that align with anomalies detected by other single-method observations, yet offers more comprehensive insights. To illustrate this, we focus on two examples: the *M*7.0 Aketao earthquake and the *M*7.1 Milin earthquake. The *M*7.0 Aketao earthquake occurred on November 25, 2016, in region No. 13. As previously noted, the model generated a false-positive prediction for an earthquake $M\ge 7$, 10 days before the actual Aketao event. To delve deeper, we’ve charted the daily $M\ge 7$ probabilities for this period. Interestingly, these probabilities show trends generally consistent with gravity observation^45^ and Load/Unload Response Ratio (LURR) theory^46^ (Fig. 4A1,A2), suggesting that what was initially identified as a false-positive may hold valuable predictive information. Specifically, SafeNet’s probability for $M\ge 7$, was in harmony with the increasing trends observed in both gravity and LURR data leading up to the Aketao event. The model not only detected the initial fluctuating stress build-up process detected by LURR but also mirrored the period of seismic calm detected by gravity measurements before the earthquake, reflected SafeNet’s sensitivity to the complexities of seismic processes.

The model’s predictive strengths were further highlighted by the *M*7.1 Milin earthquake on November 18, 2017, in region No. 81. SafeNet’s $M\ge 7$ probability trends align remarkably well with observed anomalies in thermal infrared and geomagnetic data^47^ (Fig. 4B1,B2). It is worth noting that SafeNet’s probability predictions captured anomalies that would be missed using a single observational method, providing a comprehensive pre-seismic pattern. The two earthquake events analyzed suggest that by integrating multimodal data, our model may capture a seismogenic process that more closely to the event’s essence. This also explains why observational data such as gravity, infrared, and geomagnetic measurements, though not included in our input, still align with the probability shifts in our results.

**Figure 5:**
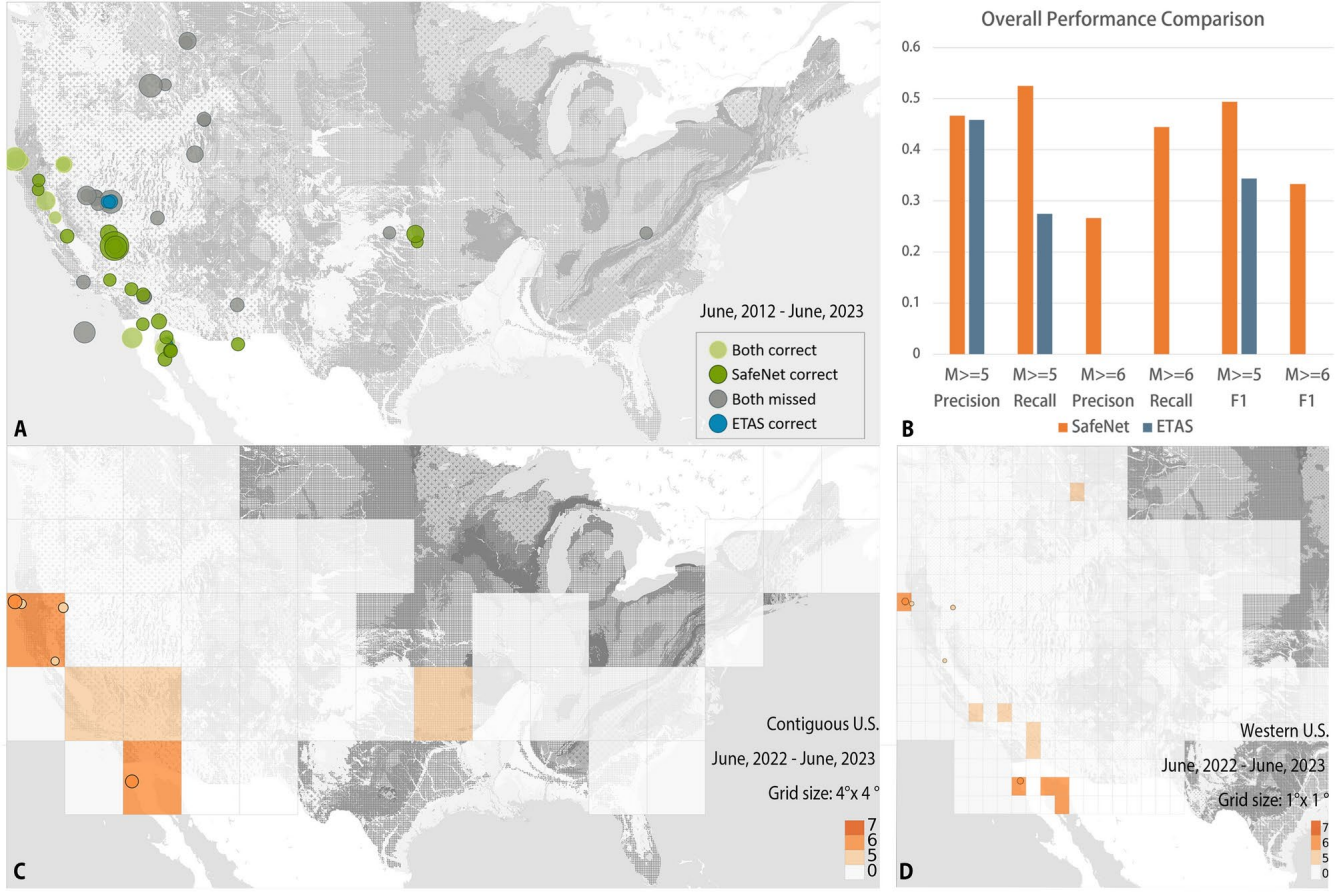
Results for the contiguous and western U.S. (**A**) Comparative Analysis: SafeNet vs. ETAS Predictions for $M \ge 5$ Events, June 2012 - June 2023, grid size: $4^{\circ }\times 4^{\circ }$. SafeNet Correct: SafeNet predicted the event correctly; ETAS did not. Both Correct: Both SafeNet and ETAS successfully predicted the event. Both Missed: Neither SafeNet nor ETAS predicted the event. ETAS Correct: ETAS predicted the event correctly; SafeNet did not. (**B**) Overall performance comparison between SafeNet and ETAS for Contiguous U.S., using a grid size of $4^{\circ }\times 4^{\circ }$. Among 40 $M\ge 5$ regions, ETAS recalled 11, with no accurate predictions for $M\ge 6$ events (it predicted 2), whereas SafeNet successfully identified 21, including 4 out of 9 regions with $M\ge 6$ earthquakes. (**C**,**D**) Prediction Results for June 25, 2022 - June 25, 2023, with different grid sizes, both successfully predicted two $M\ge 6$ earthquake regions. Maps were plotted using GMT 6^32^. See Methods for details.

### Transfer learning to the United States

The dataset used for training SafeNet does not contain any data from the Contiguous United States, making it an ideal place to test the generalization of our model. The research area spans longitude from $-125^{\circ }$ to $-65^{\circ }$ and latitude from $29^{\circ }$ to $49^{\circ }$. It contains 43 regions, each measuring $4^{\circ }\times 4^{\circ }$, selected according to the American hazard map, where more than two damaging earthquakes are expected to occur in 10,000 years^48^. The catalog was from June 18, 1998, to June 25, 2023, sourced from the U.S. Geological Survey (USGS). Given the seismic differences between the U.S. and China, we fine-tuned SafeNet using U.S. multimodal input from the first 3 years (June 18, 2008, to June 25, 2010).

The test then taken on 2012 to 2023 period. The complete set of annual predictions can be found in Figs. S9 and S10. From the overall perspective shown in Fig.  5A, B, both our model and ETAS efficiently extract spatio-temporal features; however, SafeNet’s design allows it to recall more big earthquakes. Specifically, within these 11 test years, a total of 40 out of 473 regions experienced $M\ge 5$ seismic events. The ETAS model recalled 11 of these regions, whereas our SafeNet model successfully identified 21 regions. For $M\ge 6$ regions, ETAS predicted 2, neither of which were correct, whereas our model correctly recalled 4 of the 9 regions. As shown in Fig. 5A, the events that can only be predicted by SafeNet are distributed across diverse regions, representing the model’s comprehensive improvement across different seismic backgrounds. Furthermore, all SafeNet predictions were inferred under a minute using a single V-100 GPU.

In a more localized study focusing on the western U.S., for longitudes from -$125^{\circ }$ to -$95^{\circ }$, with a $1^{\circ }\times 1^{\circ }$ grid system encompassing 5016 regions (456 regions across 11 years), SafeNet successfully predicts seismic events with $0\le$ $M\le 5$ with an accuracy of 97.35%. Furthermore, SafeNet retains its ability to recall $M \ge 6$ events in such detail grid (Fig. 5C, D). The results from the Contiguous U.S. and Western U.S. demonstrate how SafeNet can be applied at varying spatial resolutions to explore regional seismicity. By integrating additional information from surrounding regions at different spatial scales, our approach overcomes the limitations of existing models and enables more comprehensive predictions.

## Discussion

Both China and the U.S. experience frequent seismic activity across their vast territories, making them ideal places for testing large-scale $M\ge 5$ earthquake forecasting models. Among the 13 baseline models evaluated, SafeNet demonstrated remarkable predictive capabilities for $M\ge 5$ earthquakes. Furthermore, the seismic background between two countries are significantly different, which highlights SafeNet’s robustness. Using two distinct grid-size, three-year datasets from the US for fine-tuning and testing on their most recent eleven-year datasets effectively demonstrates the SafeNet’s transferbility and and its ability to integrate information across multiple scales. This experiment also shows the potential of SafeNet for earthquake prediction in regions with limited data or expertise.

However, it is important to acknowledge that the model serves as a reference for experts in regional seismic hazard assessment, particularly for conducting annual earthquake hazard assessments at the national level, rather than as a tool for precise earthquake prediction in real-world applications. Although the accuracy of the model shows potential, there remains considerable room for improvement. Enhancing catalog indicators by optimizing combinations tailored to specific forecasting objectives is a key focus to advance accuracy. In addition, incorporating a wider range of observational data (seismic, geodetic, geo-electric, etc.) could improve both the precision and reliability of forecasts. Integrating uncertainty evaluations is also crucial, as it would provide valuable insight to guide decision-making processes. Utilizing larger-scale datasets, such as global datasets, for pre-training represents a potential step toward improving model performance. This need for improvement is underscored by the limitations of relying on a single model, as seen in events predicted exclusively by ETAS (represented by blue points in Fig. 5A), which highlight the unique strengths and weaknesses of different forecasting methods. This emphasizes the necessity of adopting a diversified modeling strategy, which can provide a more comprehensive understanding of the mechanisms of earthquakes. Furthermore, the increasing demand for interpretability in artificial intelligence is driving a shift toward hybrid frameworks that combine transparent “glass-box” AI architectures with physical laws^19,31,49,50^. Crucially, our magnitude probability outputs (Figs. S3–S7) are structured to interface with regional demographic and infrastructure data. This integration-a logical next step-would enable intermediate-term socioeconomic impact assessments at the national scale, directly supporting disaster mitigation planning.

## Methods

### Multimodal geologic and seismic map

Figure 1 demonstrates this study formulates the input data as a multimodal geologic and seismic map, comprising comprehensive spatio-temporal information derived from multimodal sources. At time *t*, the multimodal map is represented as: $\mathbb {M}_t=\{M_{i,j}|i=t-(T-1),t-(T-2),...,t; j=1,2,...\}$, $M_{i,j}=\{m_{i,j,k}, d_{i,j}, c_{i,j}|k=1,2,...\}$, where $M_{i,j}$ denotes multimodal data in $j-th$ region between times $i-1$ and *i*, *T* denotes time range of multimodal data. $m_{i,j,k}$ denotes $k-th$ map of various geographic maps which can be flexibly extended, $d_{i,j}$ denotes distribution map of earthquakes, $c_{i,j}$ denotes earthquake catalog features, with potential for future expansion to encompass diverse observational data features.

### Problem formulation

Given a set of $\{\mathbb {M}_t|t=0,1,2,... ,T-1\}$ as the input of earthquake prediction. The prediction output is represented as: $\mathbb {F}_{t+1}=\{F_{t+1,j}|j=1,2,...\}$, where $F_{t+1,j}$ denotes the predicted maximum earthquake magnitude that likely to occur in $j-th$ region from time *t* to time $t+1$. Such output encapsulates the time, the location, and the magnitude range. The aim is to build a deep learning method $\mathcal {M}$, capable of effectively modeling the input ${\mathbb {M}_t}$ and predicting $\mathbb {F}_{t+1}$.

### Data process

**Data preprocessing of maps.** In the context of the Chinese dataset, initially, two types of geologic maps and 18,584 annual earthquake distribution maps were produced. The lithology map, sourced from *Generalized Geology of the Far East* by USGS^51^, detailing rock’s geologic age and type^52^. Owing to its rich, color-coded information, the map retained its three-dimensional RGB channels. The fault map, depicting the locations of China’s major faults and active tectonic, was created using data from the National Earthquake Data Center^53^. Subsequently, the fault map was converted into a single greyscale channel to conserve memory. In the earthquake distribution maps, varying circle sizes were employed to represent different earthquake magnitudes. Consequently, each earthquake distribution map at time *t* encapsulated the location and magnitude information of earthquakes from $t-1$ to *t*, where $t-1$ represents the year before *t*. The distribution maps were also grayscaled. These two geologic maps and a distribution map were integrated daily into a five-channel array and divided into $4^{\circ }\times 4^{\circ }$ regions via coordinate inversion. Each region was subsequently downscaled to a size of $50\times 50$ pixels to increase calculation efficiency. The preprocessing steps for the U.S. dataset are similar, with both the lithology map^54^ and fault map^55^ derived from USGS. All geological figures were plotted using GMT 6^32^.

**Data preprocessing of catalog.** In total, 282 seismic features were extracted (Table 1) daily for each fine-grained region and whole area from the catalog. Aiming to capture the seismicity of each region comprehensively, features were both estimated for the most recent 100 events gathered from the beginning to the present day^21^ and that of events occurring within the past year. These 282 dimensions comprise three primary types of indicators. First, time-related indicators, such as time difference and lunar date. The time difference features reveal the time interval distribution properties of the earthquakes. Regarding lunar date properties, the model accounted for solid tide modulation influences, which has a strong association with earthquake nucleation^56,57^. Secondly, location-related indicators such as depth, latitude, and longitude. Depth attributes assisted in reconstructing the 3D seismogenic environment. Latitude and longitude features are indirectly gathered by dividing regions during feature extraction. Lastly, magnitude-related indicators, including magnitude and attributes related to the Gutenberg-Richter relationship. Magnitude attributes directly reflect seismic activity and help the model predict magnitude ranges. The slope of the relationship between earthquake magnitude and the log of earthquake frequency was defined by the Gutenberg-Richter relationship^58^. Incorporating this relationship facilitated the integration of magnitude predictions with seismic activity and distribution analysis. Following feature extraction, Min-Max Normalization was employed.

### Evaluation metrics

Our evaluation strategy for the earthquake prediction model involves a comprehensive suite of metrics, meticulously chosen to assess the model’s performance across various earthquake magnitude categories. This evaluation is vital for understanding the model’s practical utility in seismology.

#### Earthquake magnitude classes

Magnitude categories are defined as $[0 \le M < 5]$, $[5 \le M < 6]$, $[6 \le M < 7]$, and $[M \ge 7]$. These categories enable targeted evaluation of the model’s predictive accuracy within specific earthquake magnitude ranges.

**Metric definitions and calculation context** In assessing the model, we utilize F1, precision, and recall for each magnitude category. The definitions of True Positives (TP), True Negatives (TN), False Positives (FP), and False Negatives (FN) are adapted to the specific context of earthquake prediction.

- **True Positives (TP)**: Occurrences where the model correctly predicts the largest earthquake within a specified magnitude range for the next year in a region.
- **False Positives (FP)**: Instances where the model incorrectly predicts an event within that magnitude range.
- **False Negatives (FN)**: Instances where the model fails to predict an actual event of such magnitude.
- **True Negatives (TN)**: Occurrences where the model correctly predicts the absence of an earthquake within a specified magnitude range.

#### Accuracy

Accuracy is defined as the ratio of correctly predicted instances (both TPs and TNs) to the total number of instances.

$$\begin{aligned} \text {Accuracy} = \frac{\text {Number of Correct Predictions}}{\text {Total Number of Predictions}} \end{aligned}$$

Mainly reflects performance for $0 \le M < 5$, due to its higher frequency in the dataset.

#### Macro F1 score

$$\begin{aligned} \text {Macro F1} = \frac{1}{N} \sum _{i=1}^{N} F1_i \end{aligned}$$

Here, *N* denotes the number of magnitude classes, and $F1_i$ is the F1 score for the $i^{th}$ class. The Macro F1 score is crucial for assessing model performance equitably across all categories, particularly when high magnitudes are less represented in the dataset.

**Metric formulas:** The calculation of each metric is tailored to reflect the model’s performance accurately in predicting earthquakes across different magnitude ranges.

$$\begin{aligned} & \text {F1} = 2 \times \frac{\text {Precision} \times \text {Recall}}{\text {Precision} + \text {Recall}}\\ & \text {Precision} = \frac{\text {True Positives}}{\text {True Positives} + \text {False Positives}}\\ & \text {Recall} = \frac{\text {True Positives}}{\text {True Positives} + \text {False Negatives}} \end{aligned}$$

The F1 score, a harmonic mean of precision and recall, offers a balanced perspective on the model’s accuracy and reliability in forecasting seismic events of varying magnitudes. Precision provides insights into the model’s ability to correctly identify significant earthquakes, and recall assesses its capability to detect as many relevant instances as possible.

**Special consideration for high-magnitude earthquakes:** Given the rarity and significant impact of higher-magnitude earthquakes, we merge categories for focused analysis:

- For $M \ge 5$: Merging $[5 \le M < 6]$, $[6 \le M < 7]$, and $[M \ge 7]$.
- For $M \ge 6$: Merging $[6 \le M < 7]$ and $[M \ge 7]$.

This aggregation is crucial for a robust analysis of the model’s capability in predicting more severe seismic events, which, though infrequent, carry significant impacts.

$$\begin{aligned} & \text {Precision}_{M \ge 5} = \frac{\text {True Positives}_{M \ge 5}}{\text {True Positives}_{M \ge 5} + \text {False Positives}_{M \ge 5}}\\ & \text {Recall}_{M \ge 5} = \frac{\text {True Positives}_{M \ge 5}}{\text {True Positives}_{M \ge 5} + \text {False Negatives}_{M \ge 5}}\\ & \text {Precision}_{M \ge 6} = \frac{\text {True Positives}_{M \ge 6}}{\text {True Positives}_{M \ge 6} + \text {False Positives}_{M \ge 6}}\\ & \text {Recall}_{M \ge 6} = \frac{\text {True Positives}_{M \ge 6}}{\text {True Positives}_{M \ge 6} + \text {False Negatives}_{M \ge 6}} \end{aligned}$$

**Runtime of predictions:** In Table  2, the term of “runtime of predictions” refers to the total execution time of different methods for predictions on the test dataset. As the methodology from^26^, our model generates predictions for all regions in China with a single forward pass, while ETAS predictions require numerous simulations for each.

**Rationale for metric selection:** The choice of these specific metrics aligns with our objective to provide a detailed and nuanced understanding of the model’s predictive accuracy in various seismic scenarios. The combination of general and magnitude-specific metrics ensures a comprehensive evaluation of the model’s efficacy in predicting earthquakes, addressing both frequent lower-magnitude events and critical, less frequent higher-magnitude earthquakes.

### Overall process

The pseudocode of the overall model training and applying process of this paper can be found in Algorithm 1.

**Algorithm 1:**
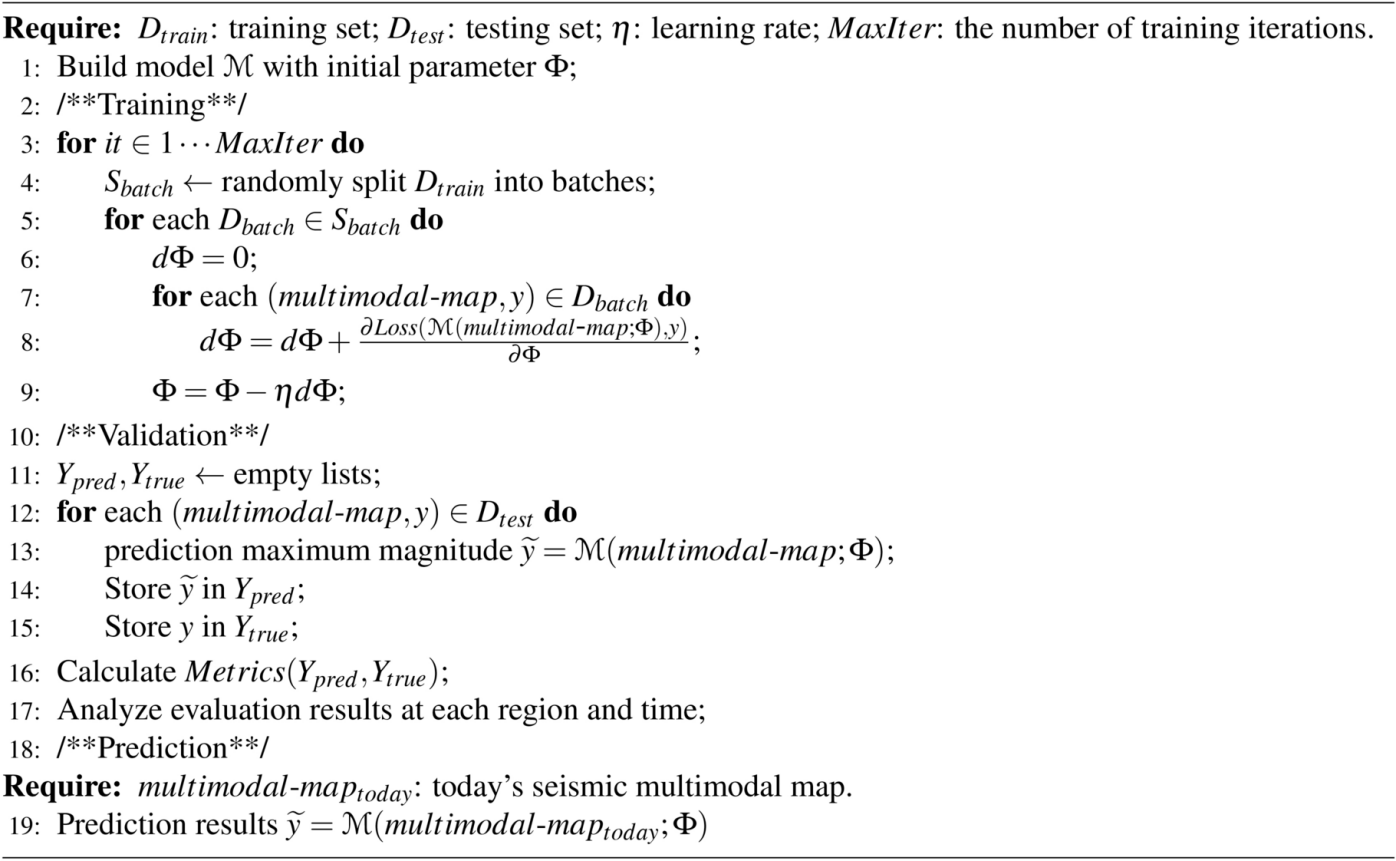
Overall Process

### Region numbering during visualization

Regions were numbered based on the distribution of faults and active tectonic blocks (the first order)^41–44^. For instance, regions No.1, No.2, and No.3 align with the direction of the Chinese Altai. Additionally, the China-Burma block, comprising only regions No.83, No.85, and No.46, spans two distinct active tectonic blocks (second order, namely Western Yunnan and Southern Yunnan). Consequently, these regions were merged with the Tibet and Northeast China blocks, respectively. This resulted in the formation of five blocks: A (West China), B (Northeast China), C (North China), D (South China), and E (Tibet).

### Earthquake prediction vision transformer

**Figure 6:**
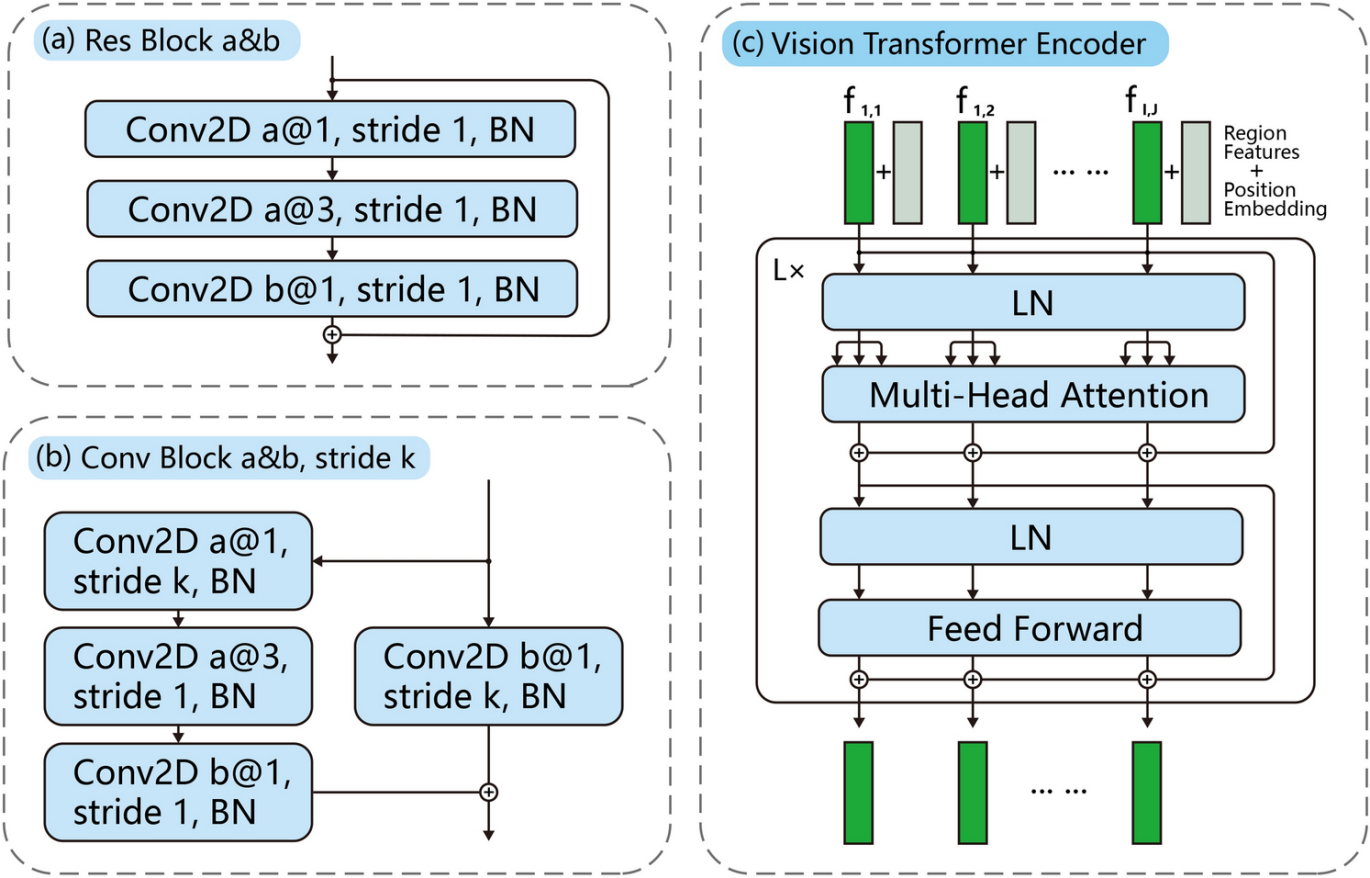
Detailed structures in SafeNet model. (**a**) Res Block: used in the Maps Embedding module for feature extraction. (**b**) Conv Block: used in the Maps Embedding module for feature extraction and dimension reduction. (**c**) Vision Transformer Encoder: used in Region-Aware Embedding module for multimodal map patches modeling.

#### Maps embedding

In the maps embedding module, we used the idea of ResNet^34^ and build the model mainly through *Res Block* and *Conv Block* to embed all information of various maps in each region. The *Res Block a&b* is the CNN-based feature extraction module with equal-dimensional input and output, which contains three convolutional layers ((a) channels with 1x1 convolution kernel, (a) channels with 3x3 convolution kernel, (b) channels with 1x1 convolution kernel) and one shortcut residual connection. The specific structure can be seen in Fig. 6a. The *Conv Block a&b* is similar to *Res Block a&b* but adds one additional convolutional layer in residual connection, which allow the output dimensions to be different from the input. This module can reduce the dimension size of feature space and the specific structure can be seen in Fig. 6b. In our experiments, we used 5 *Res Block* and 2 *Conv Block* to embed the maps in each region separately. In addition, we added one convolutional layer and max-pooling layer at the input for pattern extraction of images. And added one full connected layer and ave-pooling layer before the output to convert the image features into a feature vector that can be concatenated with other features (i.e., catalog features). The specific structure can be seen in Fig. 2(1). Formally, the *Res Block* module can be defined as:

$$\begin{aligned} y=\mathcal {F}(x,\{W_i\})+x, \end{aligned}$$

where *x* and *y* denotes the input and output vectors of the module. $\mathcal {F}(x,\{W_i\})$ represents the residual features mapping to be learned (i.e., three convolutional layers in our experiments). Similarly, the *Res Block* module can be defined as

$$\begin{aligned} y=\mathcal {F}(x,\{W_i\})+\mathcal {F}_s(x, W_s), \end{aligned}$$

where $\mathcal {F}_s(x, W_s)$ represents one projection layer (i.e., one convolutional layer in our experiments) to match the output dimensions of $\mathcal {F}(x,\{W_i\})$. Overall, the entire map embedding module can be represented as:

$$\begin{aligned} z^m_{i,j}=ME((d_{i,j}, m_{i,j,1},m_{i,j,2},\dots ),\{W_i\}), \end{aligned}$$

where $ME(\cdot )$ means maps embedding, $(d_{i,j}, m_{i,j,1},m_{i,j,2},\dots )$ are the distribution map of earthquakes and various geographic maps at time *i* and region *j*, each map contains 1 (grayscale) or 3 (RGB) image channel(s). $z^m_{i,j}$ denotes the vector representation of all image information.

#### Catalogs embedding

In the catalogs embedding module, we first extracted various statistical features from catalogs and then embedded all features as a vector representation which can combine with the vector representation of all image information. Specifically, details of each kind of catalog feature can be seen in Table 1. We used four fully connected layers to embed all features. The specific structure can be seen in Fig. 2(2). Formally, the catalogs embedding module can be defined as:

$$\begin{aligned} z^c_{i,j}=CE(c_{i,j},\{W_i\}), \end{aligned}$$

where $CE(\cdot )$ means catalogs embedding, $c_{i,j}$ denotes earthquake catalog features in the region of corresponding maps, $(d_{i,j}, m_{i,j,1},m_{i,j,2},\dots )$. $z^c_{i,j}$ denotes the vector representation of all catalog features. $z^c_{i,j}$ and $z^m_{i,j}$ can be combined together as the multimodal representation $z^t_{i,j}$:

$$\begin{aligned} z^t_{i,j}=[z^c_{i,j}\ z^m_{i,j}], \end{aligned}$$

which fuse multimodal information at each time and each region. Furthermore, we need to model the time pattern and spatial correlation of seismic multimodal information in the following modules.

#### Time-aware embedding

In the time-aware embedding module, we first reshaped the set of representations $\{z^t_{i,j}\}$ to the time series in each fine-grained region:

$$\begin{aligned} s_{j}=[z^t_{t-(T-1),j}, z^t_{t-(T-2),j}, \dots , z^t_{t,j}]. \end{aligned}$$

Then, we adopted the LSTM network to embed time-aware information from multimodal representations, formally:

$$\begin{aligned} & s'_{j}=LN(BN(s_{j})),\\ & z_{j}=LSTM(s'_{j},W_i), \end{aligned}$$

where $BN(\cdot )$ means batch normalization, $LN(\cdot )$ means layer normalization, and $LSTM(\cdot )$ means long short-term memory layer which can aggregate time series kind of input into vector representations. The specific structure can be seen in Fig. 2(3). In this way, we obtained a comprehensive representation containing seismic multimodal temporal information within each region.

#### Region-aware embedding and prediction

Based on the comprehensive seismic information representation in each region, we further modeled the spatial information around all regions. Regarding each region’s representation as an embedded patch of the image, the vision transformer module outputs region-aware embedding in each fine-grained region considering all other regions. The specific structure can be seen in Figs. 2(4) and  6c. Specifically, in the vision transformer encoder module, we further considered the characteristics of the spatial correlation of seismic information. When calculating the attention score map, we combined a mask map that decays with the spatial distance. Therefore, when our model predicts the earthquake in one region, this module can pay more attention to information about the surrounding regions. Formally, our masked self-attention can be defined as:

$$\begin{aligned} & \{a^m_{i,j}\}=\{a_{i,j}\cdot m_{i,j}\},\\ & m_{i,j}=cos\Bigg (\frac{dist(i,j)}{L_r \cdot D_{max}}\frac{\pi }{2}\Bigg ),\\ & dist(i,j)=max(|latitude_i-latitude_j|,|longitude_i-longitude_j|), \end{aligned}$$

where $a_{i,j}$ denotes the original attention score of region i to region j, $m_{i,j}$ denotes the mask map for assigning the prior bias of spatial information, and $a^m_{i,j}$ denotes the masked attention score. Meanwhile, $m_{i,j}$ is implemented by cosine function, *dist*(*i*, *j*) calculates the distance between region i and region j, $D_{max}$ is the radius parameter generally greater than the furthest distance, $L_r$ is the length of a fine-grained region, $(latitude_i, longitude_i)$ and $(latitude_j, longitude_j)$ are the latitude and longitude coordinates of region *i* and region *j*. We set $D_{max}$ as 15 and $L_r$ as $4^{\circ }$ in our experiments. This kind of mask map decays slowly in most regions and can not unduly affect the wide-area information-associated ability of the self-attention mechanism. Then, the overall region-aware embedding & prediction module can be defined as:

$$\begin{aligned} & \{z'_j\}=MVTE(\{z_j\},\{W_k\}),\\ & y_j=softmax(FC(LN(z'_j))), \end{aligned}$$

where $MVTE(\cdot )$ means masked vision transformer encoder, $FC(\cdot )$ means fully connected layer, $\{z_j\}$ denotes the set of all $z_j$, and $y_j$ denotes the final prediction results at region *j*. The results can indicate the largest magnitude of the earthquake that will occur in the next year in each region. Additionally, the time can be flexibly changed by the training label and the time interval of the dataset.

### Network configuration

Details of the network configurations are presented in Table 3. The model’s output was configured to predict maximum earthquake magnitudes into four categories: $[0 \le M < 5]$, $[5 \le M < 6]$, $[6 \le M < 7]$, and $[M \ge 7]$. The models were developed using the TensorFlow framework^59^ and optimized using the Adam Optimization Algorithm^60^. We trained 126 epochs with a learning rate of $3\times 10^{-5}$, a batch size of 4, and a dropout of 0.3. And we fine-tuned for 25 epochs on the last 20 years of the Chinese training dataset with a learning rate of $2\times 10^{-5}$. Given the seismic differences between the U.S. and China, we fine-tuned SafeNet using U.S. multimodal input from the first 3 years (June 18, 2008, to June 25, 2010) applying a learning rate of 0.001. **Platform**: All models were training/testing on a single NVIDIA A100 80 GB GPU, supported by Intel Xeon Scale 8358 CPUs.

**Table 3:** The network configurations.

Name	Value	Name	Value
Maps embedding size	32	Catalogs embedding size	32
Res blocks	[(16 &64)*2, (32 &128)*3]	Conv blocks	[16 &64, 32 &128]
Input Conv2D	32@7	Catalogs embedding MLP	[32, 32, 32, 32]
LSTM units	512	Transformers depth	3
Transformers heads	8	Head size	512
Feed forward	[1024, 512]		

### Baseline methods

Our baseline methods for earthquake prediction:

- Classic machine learning models. This group includes Linear Regression (LR), Support Vector Machine (SVM), Gradient Boosting Decision Tree (GBDT), Random Forest(RF), and Boosting. Since these methods process the structured feature vectors of fixed size, we used our 282-dimension catalog features as input. In addition, RF and Boosting were summarized in^21^ for earthquake prediction.
- Deep learning models. This group including DNN, RNN, PRNN, LSTM, Transformer, and CNN-BiLSTM with attention (CNN-BiLSTMA). RNN and PRNN also were summarized in^21^ for earthquake prediction. DNN, LSTM, and Transformer are popular sequence modeling models, which are suitable for earthquake prediction. CNN-BiLSTMA was a typical mixed model designed for earthquake prediction^24^.
- Bayesian Spatio-temporal ETAS Model. As a crucial baseline for our study, we implemented two variants of the Bayesian Spatio-temporal Epidemic-Type Aftershock Sequence (ETAS) model^39^: the ‘Proposed Method’ and the ‘Fast Method’, referred to in our paper as ‘ETAS’ and ‘Fast ETAS’, respectively. The 4$^{\circ }$ $\times$ 4$^{\circ }$ grid size for regional analysis was chosen to align with SafeNet’s grid, ensuring comparability and granularity suitable for ETAS analysis. For predicting purposes, we focused on the possibility of earthquakes with magnitudes (*m*) equal to or larger than 5, 6, and 7 in its whole region, denoted as $P(M \ge m)$^39^. A probability threshold of $P \ge 0.5$ was established, consistent with most machine learning classification models. Due to some regions lacking adequate data for ETAS calculations, we restricted our model’s results to regions with sufficient data for reliable ETAS calculations, ensuring a fair and direct comparison of model effectiveness. Calculations were performed using an Intel Xeon Scale 8358 CPU.

As shown in Table S2, we also disabled some parts of SafeNet to show their effectiveness, including four parts:

- “SafeNet w/o Maps”, where we disabled the maps embedding module. The model only used catalog features.
- “SafeNet w/o Catalogs”, where we disabled the catalogs embedding module. The model only used image data.
- “SafeNet w/o Time-Aware”, where we disabled the time-aware embedding module. The model only used data in one time step to predict the next time step.
- “SafeNet w/o Region-Aware”, where we disabled the region-aware embedding module. The model only used data in one region as input.

## Supplementary Information

Supplementary Information.

## Data Availability

The seismic catalogs for China and the United States, provided by the CENC and USGS respectively, are accessible at CodeOcean. All results from this paper can now be reproduced with one click at: https://codeocean.com/capsule/7316858/tree. Data for China’s major fault map is provided by the National Earthquake Data Center^53^, while the Generalized Geology of the Far East and the lithology and fault maps of the U.S. are all derived from the USGS^51,54,55^. Geological figures were plotted using the Generic Mapping Tools Version 6^32^.
