# Supplementary material for: Scalable intermediate-term earthquake forecasting with multimodal fusion neural networks
Source: Sci Rep. 2025 Mar 21;15:9748. doi: 10.1038/s41598-025-93877-7 (PMC11928507; doi:10.1038/s41598-025-93877-7)
Supplement: Supplementary file 1 — Supplementary Information. [file 41598_2025_93877_MOESM1_ESM.pdf]

Scalable Intermediate-term Earthquake Prediction with Multimodal Fusion Neural Networks

Supplementary Information

Supplementary Note 1: Related Works

Supplementary Note 2: Dataset Distribution (Figure S1 - S2)

Supplementary Prediction Results (Figure S3 - S10, Table S1)

Supplementary Experiments (Table S2 - S4)

Supplementary References

Supplementary Note 1: Related Works

Traditional Earthquake Prediction

Effective earthquake prediction can help mitigate seismic risks and enhance emergency management capabilities. For a long time, earthquake prediction has received significant attention, and scientists have conducted extensive exploration for effective earthquake prediction[1–4]. Early earthquake prediction research mainly relied on earthquake precursors, and many precursors have been reported from various severe earthquakes[5–10]. For example, Ohtake *et al.*[6] analyzed the relationship between seismicity gap and a large earthquake. Wakita *et al.*[7] regarded radon anomaly as a potential precursor of the 1978 Izu-Oshima-Kinkaid earthquake. Tsunogai *et al.*[10] reported the precursory chemical changes in groundwater before the Kobe earthquake. Although these precursors have been reported, utilizing these kinds of precursors to reliably predict earthquakes is still difficult. Wyss *et al.*[11] concluded that only 3 out of 28 precursors can be evaluated and possibly have a reliable correlation with earthquakes. Then, many researchers discussed the predictability of earthquakes[12,13], demonstrating that reliably predicting earthquakes solely based on precursors is really difficult. Meanwhile, to realize more reliable earthquake prediction, researchers have also proposed a series of earthquake laws based on seismic theories and historical observations and provided more probabilistic approaches to predict earthquakes. For example, Dahmen *et al.*[14] provided Gutenberg and Richter mathematical model to describe the relationship between earthquake magnitude and frequency of occurrences. Kannan *et al.*[15] combined the mathematical principle of Poisson's distribution and spatial connections for each earthquake zone to predict the next earthquake. Spatial connections, crucial for location prediction. Shen *et al.*[16] proposed a probabilistic earthquake prediction model based on the strain examined between the tectonic plates. Shcherbakov *et al.*[17] combined the Bayesian methods with the extreme value theory and assumed the occurrence of earthquakes. However, given the complexity of this natural phenomenon, earthquake laws remain challenging to describe by researchers.

Deep Learning Earthquake Prediction

In recent years, the deep learning method, recognized as a powerful technique in addressing intractable physical problems[18,19], has made significant progress in earthquake prediction. The development of deep learning technology offers new opportunities for capturing complex patterns from massive seismic data and achieve reliable earthquake prediction[4,20]. For example, Negarestani *et al.*[21] first attempted to use deep neural networks with two hidden layers for earthquake prediction. Then, Panakkat *et al.*[22,23] adopted a recurrent neural network to model multiple seismicity indicators for earthquake prediction. These methods are primarily based on indicators and regard deep learning methods as feature classifiers, and lack modeling of seismic laws. Earthquake catalogs often contain various latent information of earthquake patterns[20], therefore many studies have involved earthquake catalogs into deep learning modeling to solve a wide range of earthquake prediction problems[17,24–28]. For example, Asim *et al.*[25] extracted eight seismic indicators from catalogs and adopted four kinds of machine learning approaches for predicting earthquake magnitudes in Hindukush region. Wang *et al.*[24] split the study region into several sub-regions, and information about earthquakes and catalog data are counted according to each sub-region. The authors then constructed information of all regions as a vector input into the LSTM to predict each sub-region. This study attempts to model spatial correlation by feature interaction and validated the effectiveness and importance of spatial correlation. Kavianpour *et al.*[29] also considered the spatial correlation between the same sub-regions mentioned above. This work utilized CNN to model spatial correlation and regarded information of different sub-regions as different channels in CNN. However, these sub-regions remain overly large, and the approach to modeling spatial information is limited. To address this, we propose an attentive model which not only can comprehensively model the spatio-temporal information and explain the spatial correlation between different sub-regions but also can make predictions in each fine-grained sub-region. Furthermore, Devries *et al.*[26] forecasted the location distribution of aftershocks following large earthquakes. However, while most existing studies focus on one kind of seismic data and predict an earthquake in a large region, there is still a lack of an explainable method to involve multimodal seismic data and comprehensively modeling spatio-temporal patterns.

Neural Networks

Neural networks (NNs), recognized as powerful tools in solving machine learning tasks, are widely used in various data-driven modeling problems owing to their robust fitting and generalization capabilities[30–33]. The application of artificial neural network models in earthquake-related problems has also attracted wide attention, and primarily encompass three types of models: recurrent neural network (RNN), convolutional neural network (CNN), and attention-mechanism neural network. Recurrent neural network[34] focuses on the modeling of sequence data, especially the modeling of temporal information. This kind of neural network can capture sequence dependencies in iterative progress. Further, the Long Short-Term Memory Model (LSTM) [35], as a representative RNN variant, can model both the long-term and short-term memories, and provide memory control gates and memory units, to update the long-term and short-term information. A convolutional neural network (CNN) extracts image patterns through a set of convolution kernels, and expands the kernel receptive field in the deep layers[36]. This kind of method has powerful representation capabilities and parallel computing capabilities for modeling and embedding image data[37]. One of

the most representative works is the Inception series networks[38,39], which further improves the ability of image representation by using the combination of multi-scale convolution kernels and residual connection structure. Attention-mechanism neural networks have attracted wide attention due to their powerful modeling ability and interpretability. Notably, Vaswani *et al.*[40] introduced the Transformer model, modeling a series of data points solely through an attention mechanism. Meanwhile, the proposed multi-head self-attention mechanism interprets relationships between various positional data points, capture long-range interaction patterns and avoid long-range information loss. This algorithm has achieved remarkable results in natural language processing[41,42]. Recently, these networks are increasingly applied in the field of computer vision[43,44] and time series modeling[45,46]. Owing to their flexible representational capabilities and robust information association in diverse fields, multimodal models based on attention mechanisms are emerging[47,48]. In the seismic study field, there are a variety of multimodal data, which can reflect the information on earthquakes from different aspects. In this study, we integrate various neural network models to represent different aspects of information and utilize attention mechanisms to correlate information in different regions and different modalities to achieve more comprehensive and reliable earthquake prediction.

Supplementary Note 2: Data Distribution

Data Source

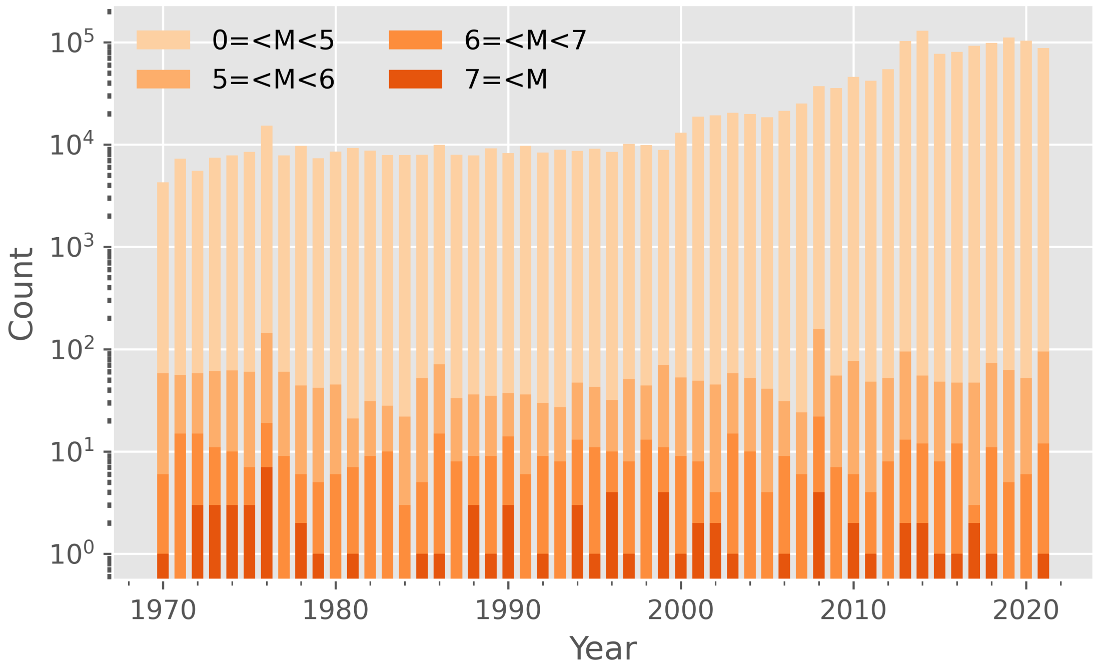

Supplementary Figure S1. Dataset distribution of each year. We plot the number of events for each magnitude level annually from 1970 to 2021. The y-axis is log-scaled. Owing to the increase in seismic networks and earthquake monitoring capability, the number of earthquakes below magnitude 5 has exponentially increased from 2000. The number of earthquakes of varying magnitudes differs significantly, resulting in extremely imbalanced classification categories. From 1970 to 2021, on average, there are 24 M≥5 earthquakes, 4 M≥6 earthquakes, and 0.6 M≥7 earthquakes per year occurring in the Chinese dataset. To improve the model's generalization ability, all regions are trained jointly, which means when there is a high-magnitude earthquake in one region, other regions will also participate in training by sharing their seismic information.

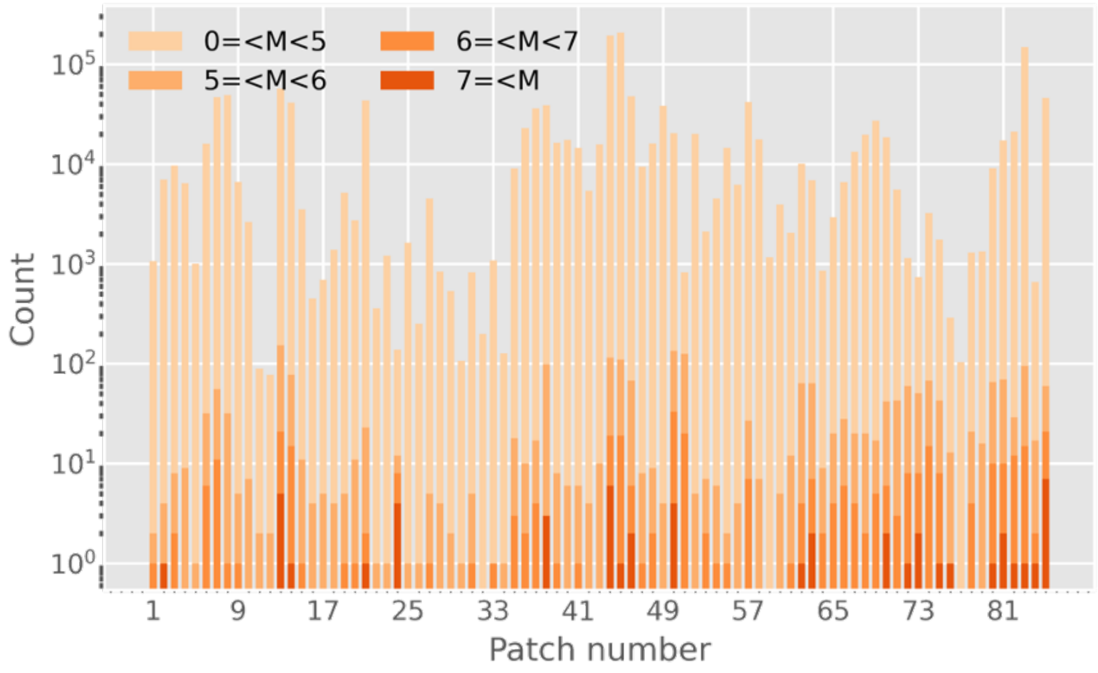

Supplementary Figure S2. **Dataset distribution of each region.** We plot the number of events for each magnitude level in each region over a span of 52 years (1970-2021) The y-axis is log-scaled. In different regions, the distribution of earthquakes varies greatly. To address this, we utilize a region-aware embedding module and geological maps.

Supplementary Prediction Results in China

Supplementary Table S1. Overall performance on binary classification. We categorize events with magnitudes $5 \leq M$ as positives, and those with $M < 5$ as negatives.

Model	Accuarcy	$5 \leq M$ Precision	$5 \leq M$ Recall	$5 \leq M$ F1	$5 \leq M$ Weighted-F1
RF*	0.5765	0.3374	0.731	0.4618	0.603
RNN*	0.6588	0.3619	0.489	0.4161	0.673
PRNN*	0.5882	0.3557	0.810	0.4944	0.613
Boosting*	0.5464	0.3312	0.810	0.4702	0.570
CNNbilstmA	0.6850	0.3924	0.4895	0.4356	0.6956
PRNN	0.6902	0.3991	0.4895	0.4397	0.6999
DNN	0.7216	0.4196	0.3158	0.3604	0.7074
Transformer	0.7229	0.4304	0.3579	0.3908	0.7139
RNN	0.7647	0.5595	0.2474	0.3431	0.7291
LSTM	0.7660	0.5647	0.2526	0.3491	0.7311
LR	0.7660	0.6038	0.1684	0.2634	0.7125
GBDT	0.7765	0.7317	0.1579	0.2597	0.7172
SVM	0.7791	0.7059	0.1895	0.2988	0.7273
RF	0.7856	0.6711	0.2684	0.3835	0.7493
Boosting	0.7948	0.646	0.3842	0.4818	0.7751
**SafeNet**	**0.8039**	0.6408	0.4789	**0.5482**	**0.7937**

* denotes the use of the baseline's original 8 indicators.

Prediction results: November 16, 2012 - November 16, 2013

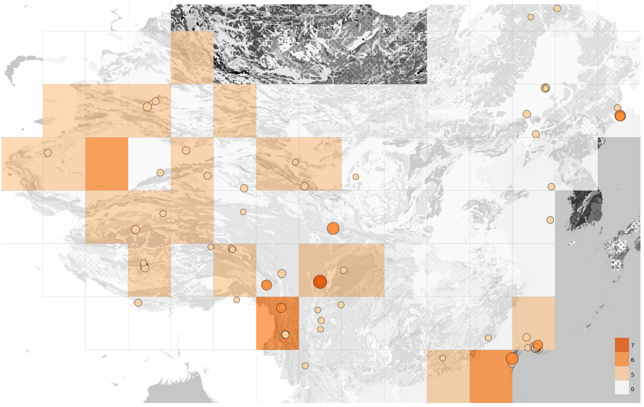

(a) prediction results for November 16, 2012 - November 16, 2013.

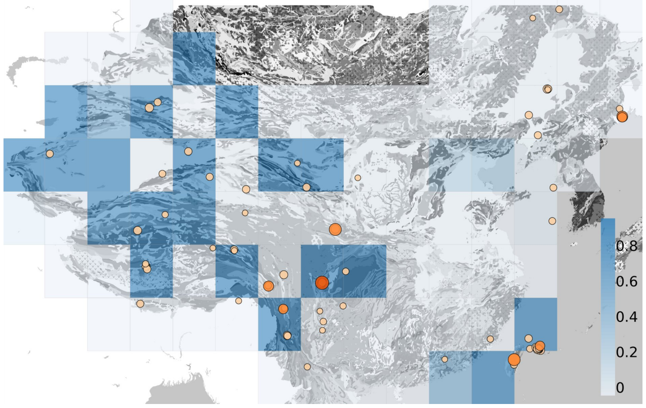

(b) Probability distribution of $M \geq 5$ for November 16, 2012 - November 16, 2013.

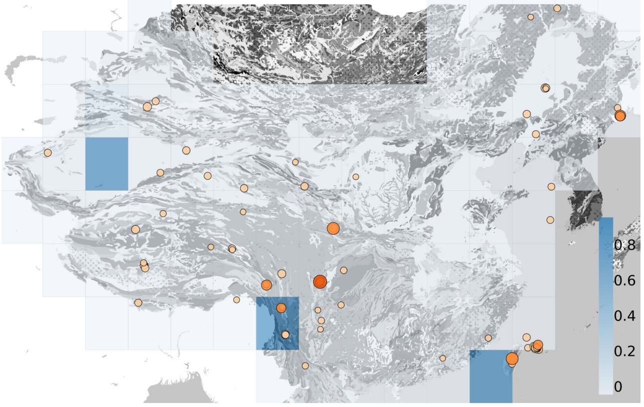

(c) Probability distribution of $M \geq 6$ for November 16, 2012 - November 16, 2013.

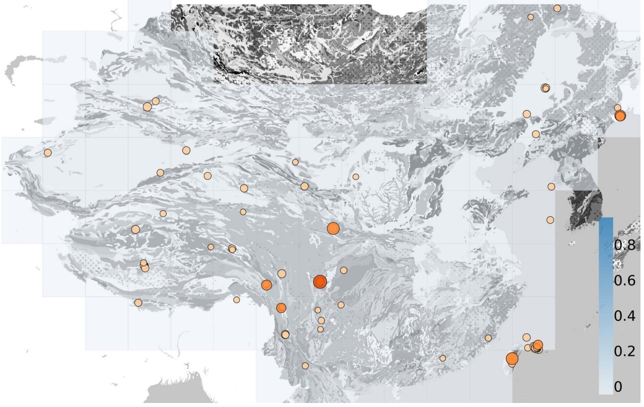

(d) Probability distribution of $M \geq 7$ for November 16, 2012 - November 16, 2013.

Supplementary Figure S3. Example of prediction results. (a)&(b) Prediction result. The colored regions indicate where SafeNet predicts earthquakes of varying magnitudes will occur, while the colored dots represent the actual recorded magnitudes of earthquakes (ground truth) (c)-(e) Detail of the probability distribution for the predictions in (a); (f)-(h) Detail of the probability distribution for the predictions in (b). Maps were plotted using the Generic Mapping Tools Version 6 (GMT6)[49].

Prediction results: November 16, 2013 - November 16, 2015

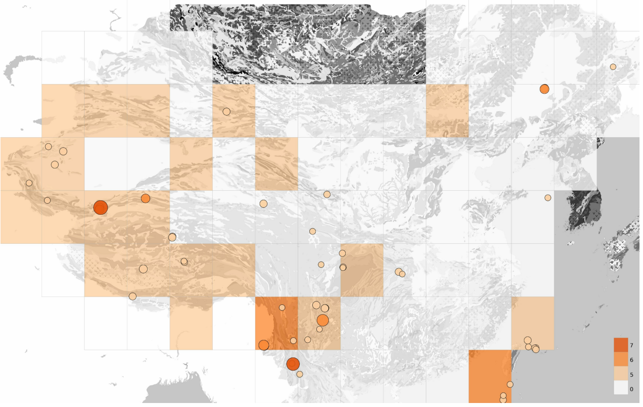

(a) Prediction results for November 16, 2013 - November 16, 2014.

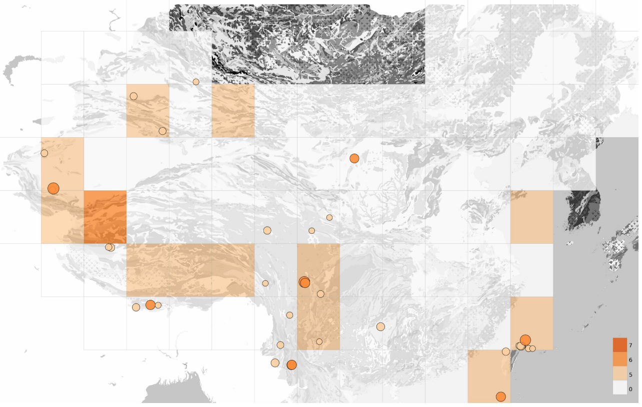

(b) Prediction results for November 16, 2014 - November 16, 2015.

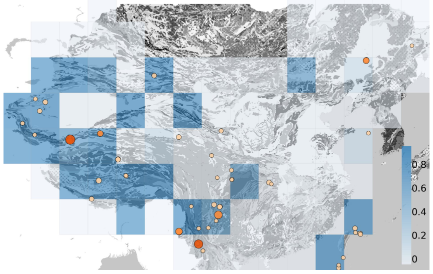

(c) Probability distribution of $M \geq 5$ for November 16, 2013 - November 16, 2014.

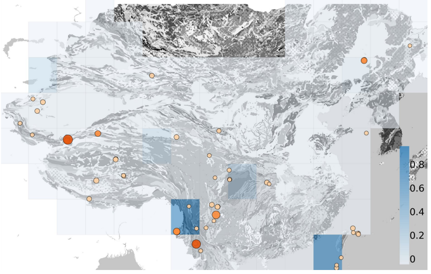

(d) Probability distribution of $M \geq 6$ for November 16, 2013 - November 16, 2014.

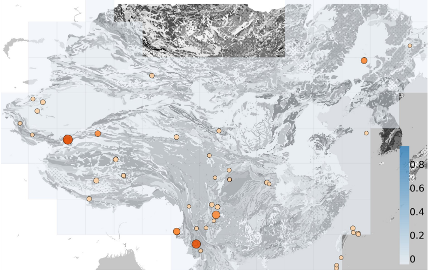

(e) Probability distribution of $M \geq 7$ for November 16, 2013 - November 16, 2014.

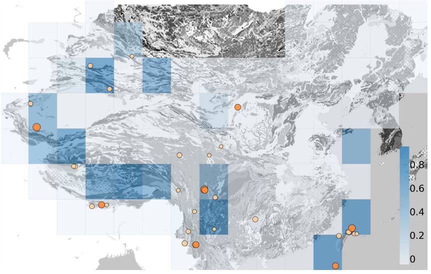

(f) Probability distribution of $M \geq 5$ for November 16, 2014 - November 16, 2015.

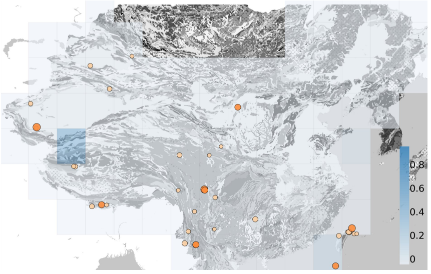

(g) Probability distribution of $M \geq 6$ for November 16, 2014 - November 16, 2015.

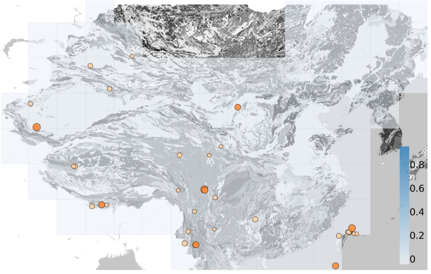

(h) Probability distribution of $M \geq 7$ for November 16, 2014 - November 16, 2015.

Supplementary Figure S4. Example of prediction results. (a)&(b) Prediction result. The colored regions indicate where SafeNet predicts earthquakes of varying magnitudes will occur, while the colored dots represent the actual recorded magnitudes of earthquakes (ground truth) (c)-(e) Detail of the probability distribution for the predictions in (a); (f)-(h) Detail of the probability distribution for the predictions in (b). Maps were plotted using GMT 6[49].

Prediction results: November 16, 2015 - November 16, 2017

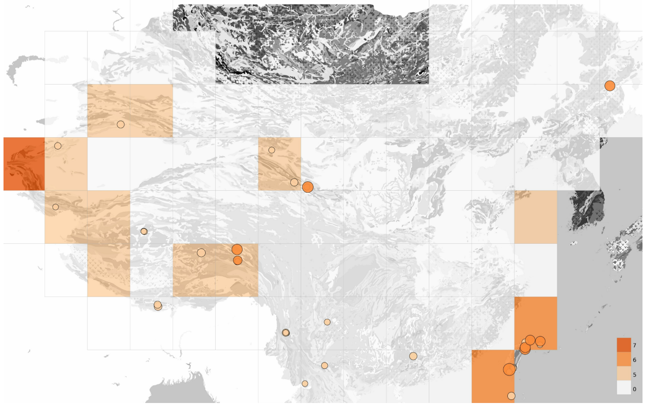

(a) Prediction results for November 16, 2015 - November 16, 2016.

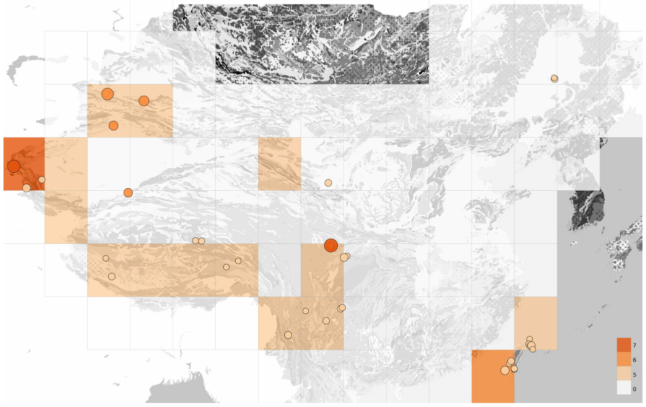

(b) Prediction results for November 16, 2016 - November 16, 2017.

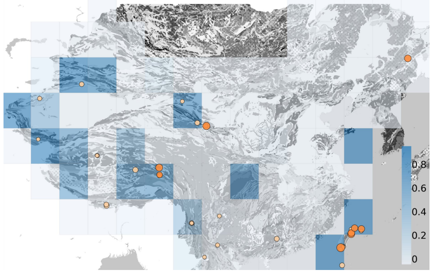

(c) Probability distribution of $M \geq 5$ for November 16, 2015 - November 16, 2016.

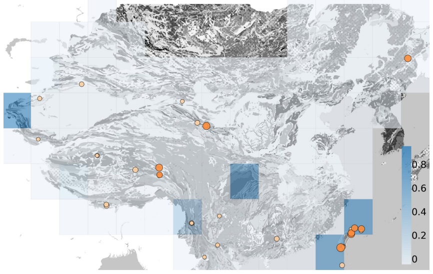

(d) Probability distribution of $M \geq 6$ for November 16, 2015 - November 16, 2016.

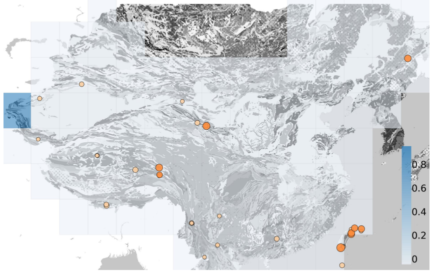

(e) Probability distribution of $M \geq 7$ for November 16, 2015 - November 16, 2016.

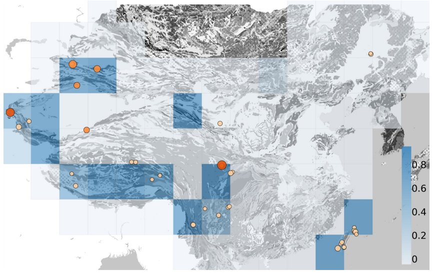

(f) Probability distribution of $M \geq 5$ for November 16, 2016 - November 16, 2017.

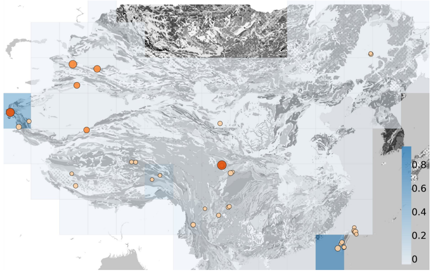

(g) Probability distribution of $M \geq 6$ for November 16, 2016 - November 16, 2017.

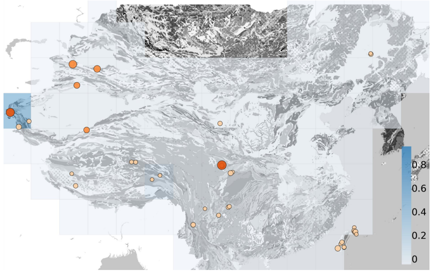

(h) Probability distribution of $M \geq 7$ for November 16, 2016 - November 16, 2017.

Supplementary Figure S5. Example of prediction results. (a)&(b) Prediction result. The colored regions indicate where SafeNet predicts earthquakes of varying magnitudes will occur, while the colored dots represent the actual recorded magnitudes of earthquakes (ground truth) (c)-(e) Detail of the probability distribution for the predictions in (a); (f)-(h) Detail of the probability distribution for the predictions in (b). Maps were plotted using GMT 6[49].

Prediction results: November 16, 2017 - November 16, 2019

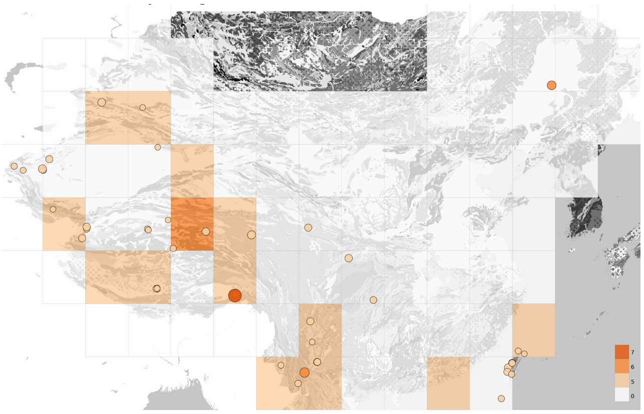

(a) Prediction results for November 16, 2017 - November 16, 2018.

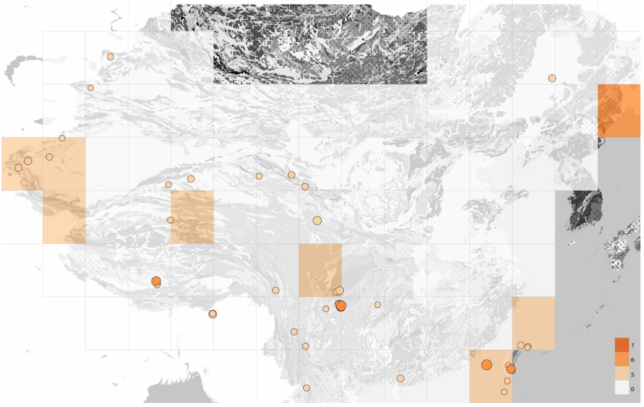

(b) Prediction results for November 16, 2018 - November 16, 2019.

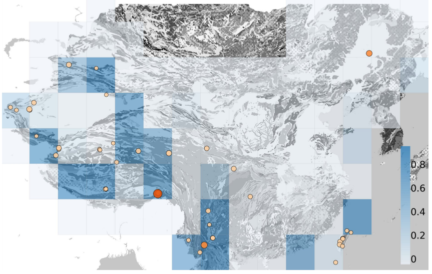

(c) Probability distribution of $M \geq 5$ for November 16, 2017 - November 16, 2018.

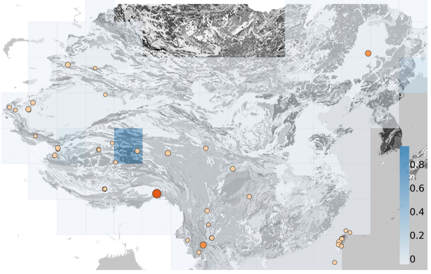

(d) Probability distribution of $M \geq 6$ for November 16, 2017 - November 16, 2018.

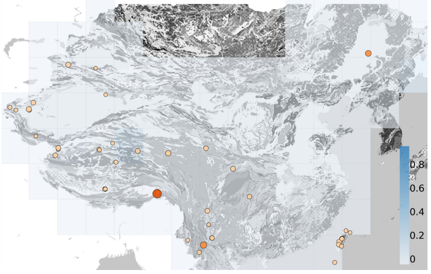

(e) Probability distribution of $M \geq 7$ for November 16, 2017 - November 16, 2018.

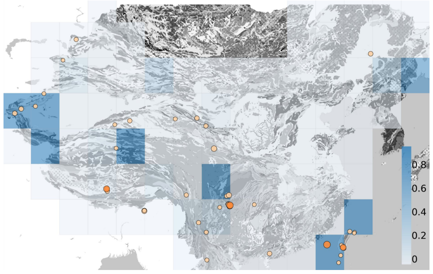

(f) Probability distribution of $M \geq 5$ for November 16, 2018 - November 16, 2019.

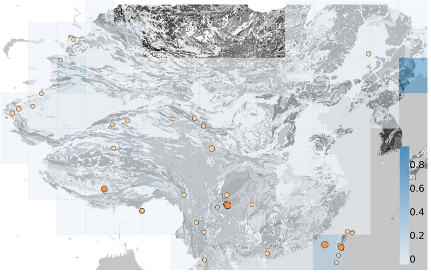

(g) Probability distribution of $M \geq 6$ for November 16, 2018 - November 16, 2019.

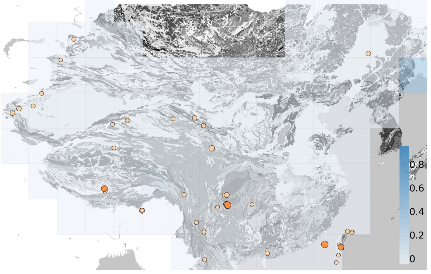

(h) Probability distribution of $M \geq 7$ for November 16, 2018 - November 16, 2019.

Supplementary Figure S6. Example of prediction results. (a)&(b) Prediction result. The colored regions indicate where SafeNet predicts earthquakes of varying magnitudes will occur, while the colored dots represent the actual recorded magnitudes of earthquakes (ground truth) (c)-(e) Detail of the probability distribution for the predictions in (a); (f)-(h) Detail of the probability distribution for the predictions in (b). Maps were plotted using GMT 6[49].

Prediction results: November 16, 2019 - November 16, 2021

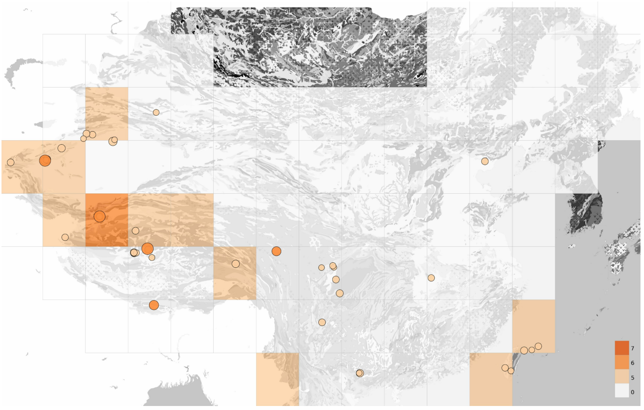

(a) Prediction results for November 16, 2019 - November 16, 2020.

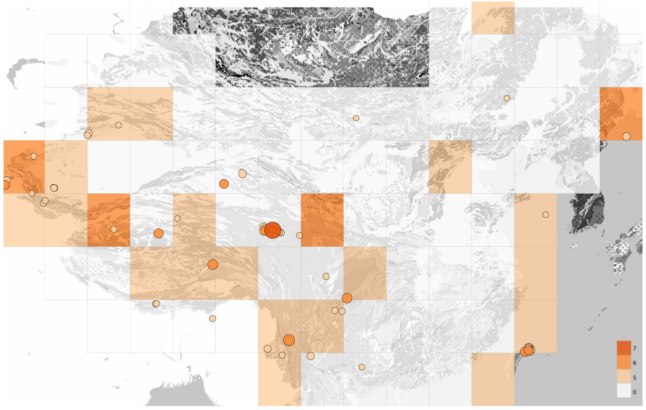

(b) Prediction results for November 16, 2020 - November 16, 2021.

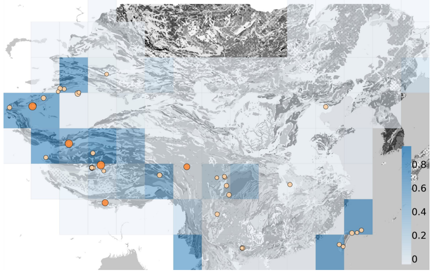

(c) Probability distribution of $M \geq 5$ for November 16, 2019 - November 16, 2020.

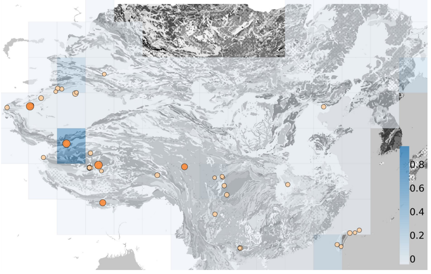

(d) Probability distribution of $M \geq 6$ for November 16, 2019 - November 16, 2020.

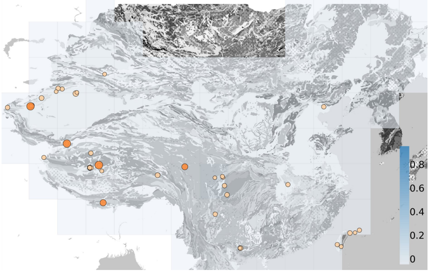

(e) Probability distribution of $M \geq 7$ for November 16, 2019 - November 16, 2020.

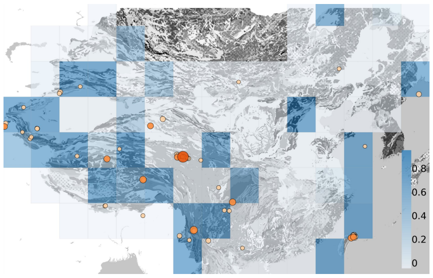

(f) Probability distribution of $M \geq 5$ for November 16, 2020 - November 16, 2021.

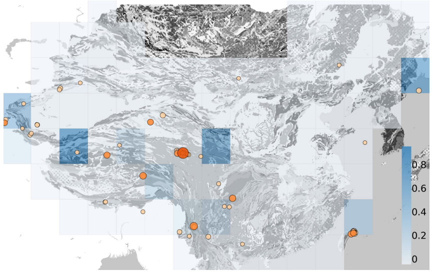

(g) Probability distribution of $M \geq 6$ for November 16, 2020 - November 16, 2021.

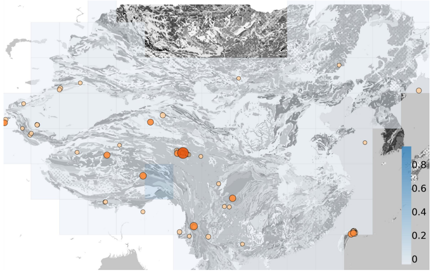

(h) Probability distribution of $M \geq 7$ for November 16, 2020 - November 16, 2021.

Supplementary Figure S7. Example of prediction results. (a)&(b) Prediction result. The colored regions indicate where SafeNet predicts earthquakes of varying magnitudes will occur, while the colored dots represent the actual recorded magnitudes of earthquakes (ground truth) (c)-(e) Detail of the probability distribution for the predictions in (a); (f)-(h) Detail of the probability distribution for the predictions in (b). Maps were plotted using GMT 6[49].

Attention distribution

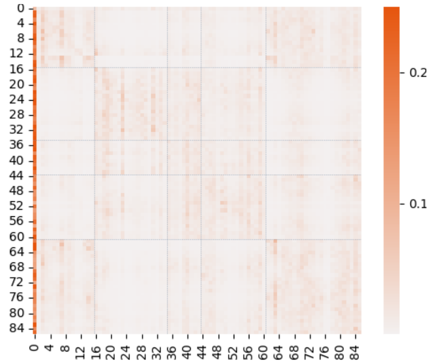

(a) Averaged attention map.

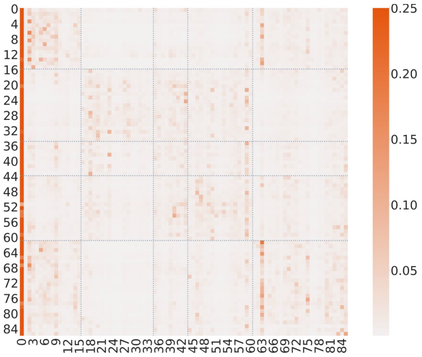

(b) Attention map of November 16, 2012 - November 16, 2013.

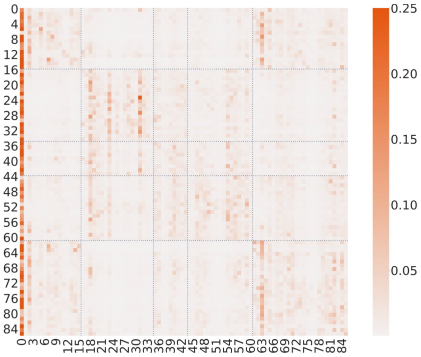

(c) Attention map of November 16, 2013 - November 16, 2014.

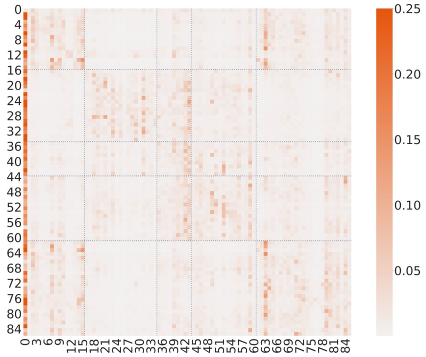

(d) Attention map of November 16, 2014 - November 16, 2015.

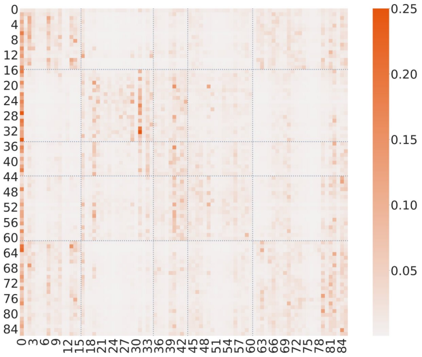

(e) Attention map of November 16, 2015 - November 16, 2016.

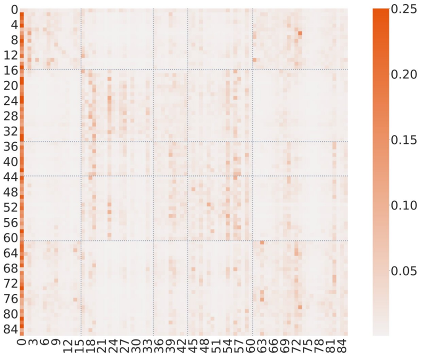

(f) Attention map of November 16, 2017 - November 16, 2018.

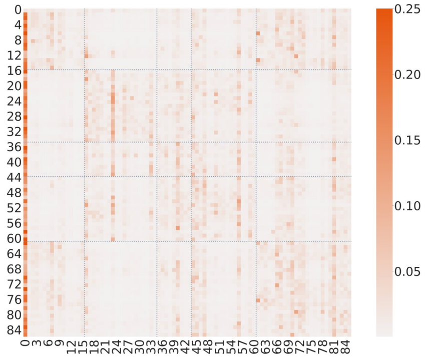

(g) Attention map of November 16, 2018 - November 16, 2019.

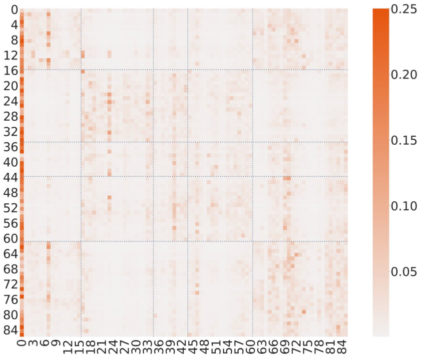

(h) Attention map of November 16, 2019 - November 16, 2020.

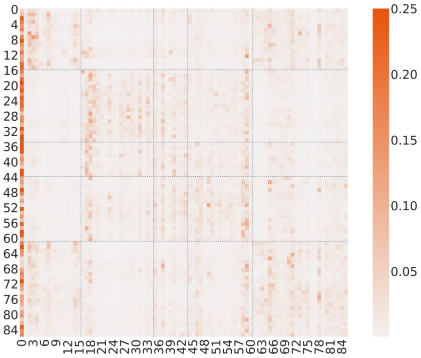

(i) Attention map of November 16, 2020 - November 16, 2021.

Supplementary Figure S8. Attention maps. (a)Averaged attention map for Nine testing years. (b)-(i) Individual attention map for each testing year. Maps were plotted using GMT 6[49].

Supplementary Prediction Results in the Contiguous United States

Prediction Results for the Conterminous U.S. Using $4^\circ \times 4^\circ$ Grid Size

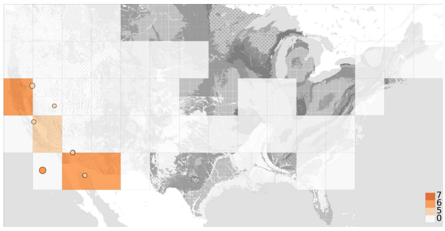

(a) June 25, 2012 - June 25, 2013

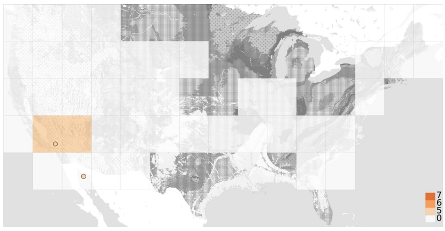

(b) June 25, 2013 - June 25, 2014.

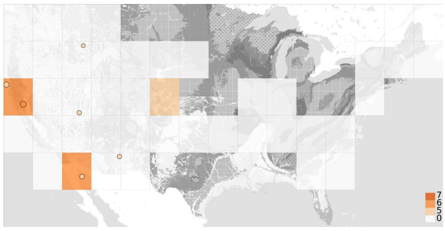

(c) June 25, 2014 - June 25, 2015.

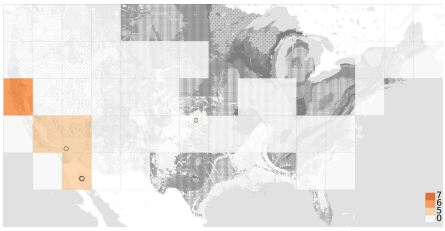

(d) June 25, 2015 - June 25, 2016.

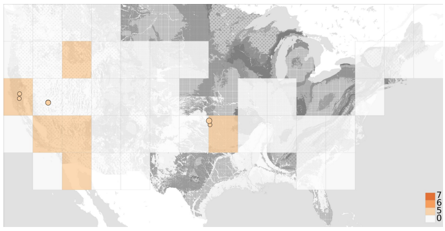

(e) June 25, 2016 - June 25, 2017.

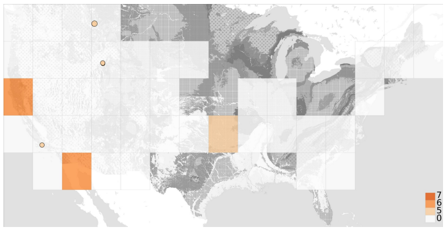

(f) June 25, 2017 - June 25, 2018.

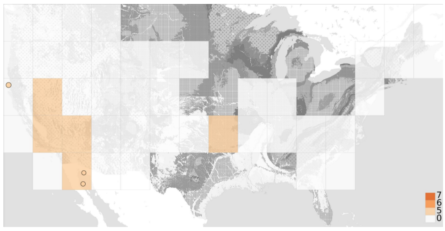

(g) June 25, 2018 - June 25, 2019.

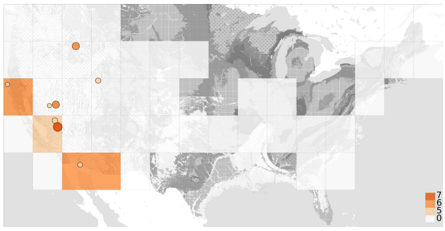

(h) June 25, 2019 - June 25, 2020.

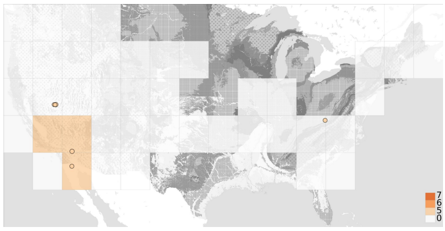

(i) June 25, 2020 - June 25, 2021.

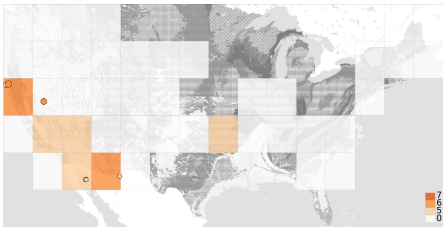

(j) June 25, 2021 - June 25, 2022.

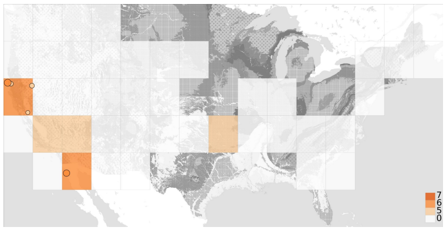

(k) June 25, 2022 - June 25, 2023.

Supplementary Figure S9. **Contiguous U.S. prediction results. Maps were plotted using GMT 6[49].**

Prediction Results for the Western U.S. Using $1^\circ \times 1^\circ$ Grid Size

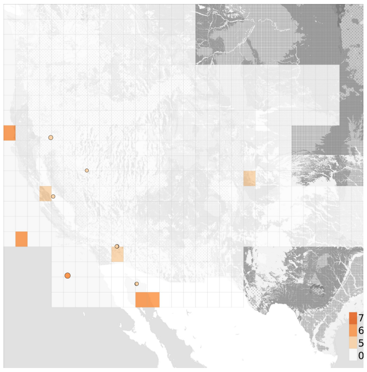

(a) June 25, 2012 - June 25, 2013

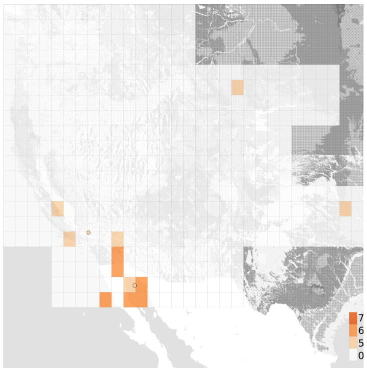

(b) June 25, 2013 - June 25, 2014.

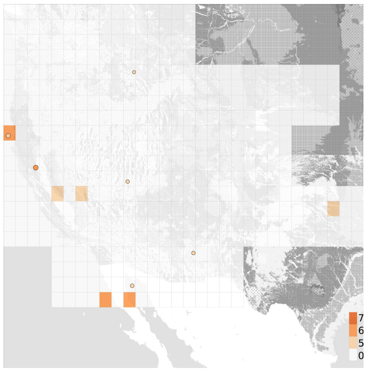

(c) June 25, 2014 - June 25, 2015.

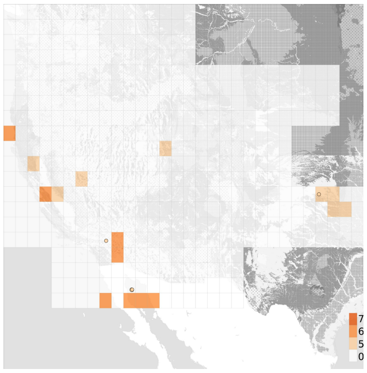

(d) June 25, 2015 - June 25, 2016.

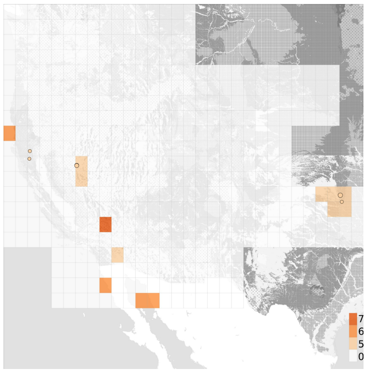

(e) June 25, 2016 - June 25, 2017.

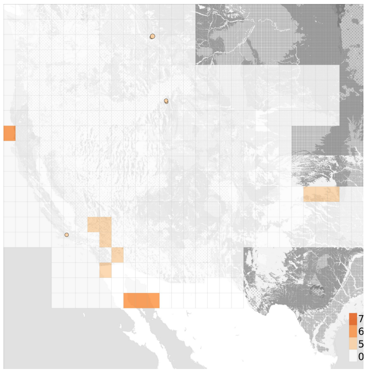

(f) June 25, 2017 - June 25, 2018.

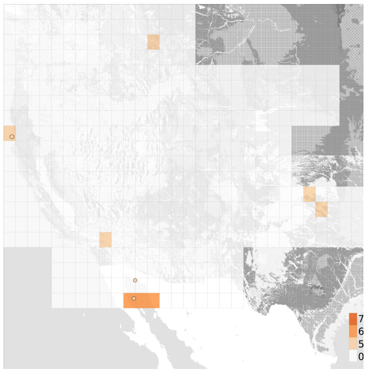

(g) June 25, 2018 - June 25, 2019.

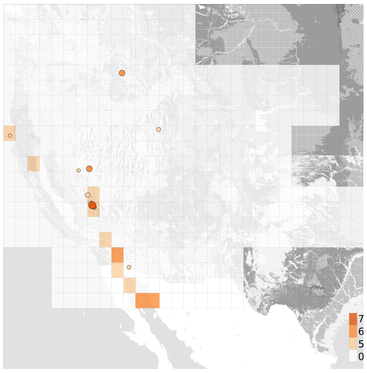

(h) June 25, 2019 - June 25, 2020.

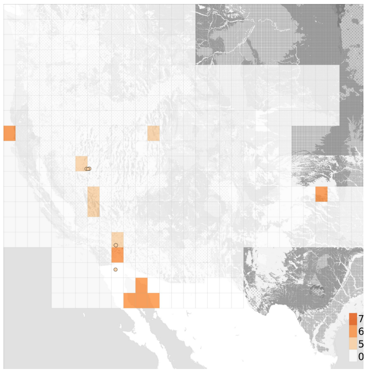

(i) June 25, 2020 - June 25, 2021.

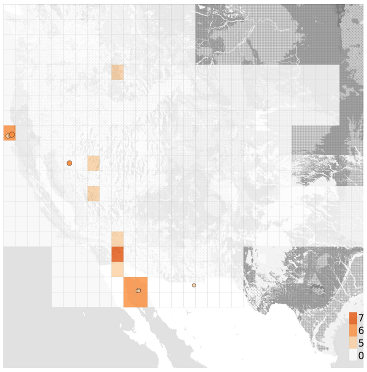

(j) June 25, 2021 - June 25, 2022.

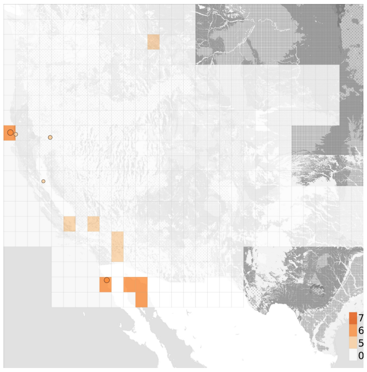

(k) June 25, 2022 - June 25, 2023.

Supplementary Figure S10. **Western U.S. prediction results. Maps were plotted using GMT 6[49].**

Supplementary Experiments

Ablation

To assess the efficacy of our model's individual modules, we conducted a series of ablation studies. These involved selectively omitting components: the Time-Aware module (excluding temporal data beyond the previous year), the Region-Aware module (limiting input to a single region's data), the Catalogs module (removing catalog features), and the Maps module (excluding map data). The results, detailed in Table S2, demonstrate a notable performance decline in the absence of any module, underscoring their collective significance in earthquake prediction. Despite these exclusions, the overall performance (Macro-F1 score) of these modified models still surpassed baseline levels, demonstrating our model's robustness. Specifically, the Time-Aware Embedding significantly aids in detecting long-term seismic patterns, which are overlooked when limited to single-year data. The Region-Aware Embedding enhances the model's ability to discern inter-regional relationships, crucial for detailed regional earthquake prediction. While the absence of catalog features does not impede the model's basic predictive capacity, their integration enhances prediction accuracy. Similarly, the omission of map data markedly reduces prediction effectiveness, confirming the value of geospatial information in earthquake prediction. The integration of earthquake catalog features with geological data enriches our understanding of seismic activity patterns across different regions.

Supplementary Table S2. Ablation experiments.

Model	Accuarcy	Macro F1	0≦ M<5			5≦ M<6			6≦ M<7			7≦ M		
			Precision	Recall	F1	Precision	Recall	F1	Precision	Recall	F1	Precision	Recall	F1
w/o Time-Aware	0.749	0.371	0.822	0.897	0.850	0.446	0.348	0.390	0.308	0.191	0.230	0.000	0.000	0.000
w/o Region-Aware	0.727	0.366	0.824	0.878	0.850	0.373	0.333	0.350	0.143	0.071	0.090	0.200	0.143	0.160
w/o Catalogs	0.743	0.380	0.830	0.889	0.850	0.464	0.362	0.400	0.179	0.119	0.140	0.091	0.143	0.110
w/o Maps	0.758	0.365	0.823	0.911	0.860	0.459	0.355	0.400	0.316	0.143	0.190	0.000	0.000	0.000
SafeNet	0.770	0.442	0.841	0.911	0.875	0.460	0.404	0.430	0.438	0.167	0.241	0.500	0.143	0.222

Time-window length experiments

To further evaluate the importance of temporal information, we explore the prediction performance with different time-window lengths. As indicated in Table S3, with the window length extending from 3 years to 10 years, years, the model's comprehensive prediction performance, as reflected by the Macro-F1 score, correspondingly improves. This indicates that our model effectively utilizes information from the long historical seismic background in predicting future earthquakes.

Supplementary Table S3. Time-window length experiments.

Model	Accuarcy	F1-macro	0≦ M<5			5≦ M<6			6≦ M<7			7≦ M		
			Precision	Recall	F1	Precision	Recall	F1	Precision	Recall	F1	Precision	Recall	F1
3year	0.741	0.361	0.823	0.890	0.850	0.390	0.340	0.360	0.350	0.167	0.220	0.000	0.000	0.000
5year	0.741	0.398	0.825	0.903	0.860	0.373	0.291	0.320	0.250	0.143	0.180	0.500	0.143	0.220
10year	0.770	0.442	0.841	0.911	0.875	0.460	0.404	0.430	0.438	0.167	0.241	0.500	0.143	0.222

Supplementary Table S4. Results of paired t-tests and Wilcoxon tests

Model	t-value	p-value	Wilcoxon p-value
Boosting	3.55603	0.00077	0.00251
DNN	2.00779	0.04950	0.04604
GBDT	4.34168	0.00006	0.00009
LR	4.38607	0.00005	0.00006
RF	2.92137	0.00502	0.00232
SVM	3.06545	0.00334	0.00120
RNN	3.62600	0.00062	0.00059
PRNN	2.91334	0.00513	0.00103
LSTM	2.43013	0.01832	0.02036
CNNbilstmA	2.10376	0.03990	0.04098
Transformer	3.18607	0.00236	0.00223
ETAS	2.20887	0.03129	0.04354

Paired t-tests and Wilcoxon tests

Besides, we conducted rigorous paired t-tests and Wilcoxon tests for each baseline method. To mitigate the impact of regions without seismic events, we concentrated on 57 regions with $M \geq 5$ events. We calculated the macro F1 score, which we consider to be the most representative evaluation metric for assessing the predictive capability of earthquake models on an imbalanced dataset, across the test years in each region. Subsequently, we performed paired t-tests and Wilcoxon tests for each baseline method in comparison with Safenet. Table S4 demonstrate that our model exhibits a significant predictive advantage ($p<0.05$) in these 57 regions that have previously experienced $M \geq 5$ earthquakes for each baseline method.

Supplementary References

1. Geller, R. J. Earthquake prediction: a critical review. *Geophysical Journal International* **131**, 425–450 (1997).
2. Kanamori, H. 72 Earthquake prediction: An overview. In *International Geophysics*, vol. 81, 1205–1216 (Elsevier, 2003).
3. Sobolev, G. A. Methodology, results, and problems of forecasting earthquakes. *Herald of the Russian Academy of Sciences* **85**, 107–111 (2015).
4. Mignan, A. & Broccardo, M. Neural network applications in earthquake prediction (1994–2019): Meta-analytic and statistical insights on their limitations. *Seismological Research Letters* **91**, 2330–2342 (2020).
5. Aggarwal, Y. P., Sykes, L. R., Armbruster, J. & Sbar, M. L. Premonitory changes in seismic velocities and prediction of earthquakes. *Nature* **241**, 101–104 (1973).
6. Ohtake, M., Matumoto, T. & Latham, G. V. Seismicity gap near oaxaca, southern mexico as a probable precursor to a large earthquake. In *Stress in the Earth*, 375–385 (Springer, 1977).
7. Wakita, H., Nakamura, Y., Notsu, K., Noguchi, M. & Asada, T. Radon anomaly: a possible precursor of the 1978 izu-oshima-kinkai earthquake. *Science* **207**, 882–883 (1980).
8. Roeloffs, E. A. Hydrologic precursors to earthquakes: A review. *Pure and Applied Geophysics* **126**, 177–209 (1988).
9. Fraser-Smith, A. C. *et al.* Low-frequency magnetic field measurements near the epicenter of the ms 7.1 loma prieta earthquake. *Geophysical Research Letters* **17**, 1465–1468 (1990).
10. Tsunogai, U. & Wakita, H. Precursory chemical changes in ground water: Kobe earthquake, japan. *Science* **269**, 61–63 (1995).
11. Wyss, M. Evaluation of proposed earthquake precursors (1991).
12. Geller, R. J., Jackson, D. D., Kagan, Y. Y. & Mulargia, F. Earthquakes cannot be predicted. *Science* **275**, 1616–1616 (1997).
13. Wyss, M. Cannot earthquakes be predicted? *Science* **278**, 487–490 (1997).
14. Dahmen, K., Ertaş, D. & Ben-Zion, Y. Gutenberg-richter and characteristic earthquake behavior in simple mean-field models of heterogeneous faults. *Physical Review E* **58**, 1494 (1998).
15. Kannan, S. Innovative mathematical model for earthquake prediction. *Engineering Failure Analysis* **41**, 89–95 (2014).
16. Shen, Z.-K., Jackson, D. D. & Kagan, Y. Y. Implications of geodetic strain rate for future earthquakes, with a five-year forecast of m5 earthquakes in southern california. *Seismological Research Letters* **78**, 116–120 (2007).
17. Shcherbakov, R., Zhuang, J., Zöller, G. & Ogata, Y. Forecasting the magnitude of the largest expected earthquake. *Nature communications* **10**, 1–11 (2019).
18. Pathak, J., Hunt, B., Girvan, M., Lu, Z. & Ott, E. Model-free prediction of large spatiotemporally chaotic systems from data: A reservoir computing approach. *Physical review letters* **120**, 024102 (2018).
19. Carleo, G. *et al.* Machine learning and the physical sciences. *Reviews of Modern Physics* **91**, 045002 (2019).
20. Beroza, G. C., Segou, M. & Mostafa Mousavi, S. Machine learning and earthquake forecasting—next steps. *Nature communications* **12**, 1–3 (2021).
21. Negarestani, A., Setayeshi, S., Ghannadi-Maragheh, M. & Akashe, B. Layered neural networks based analysis of radon concentration and environmental parameters in earthquake prediction. *Journal of environmental radioactivity* **62**, 225–233 (2002).
22. Panakkat, A. & Adeli, H. Neural network models for earthquake magnitude prediction using multiple seismicity indicators. *International journal of neural systems* **17**, 13–33 (2007).
23. Panakkat, A. & Adeli, H. Recurrent neural network for approximate earthquake time and location prediction using multiple seismicity indicators. *Computer-Aided Civil and Infrastructure Engineering* **24**, 280–292 (2009).
24. Wang, Q., Guo, Y., Yu, L. & Li, P. Earthquake prediction based on spatio-temporal data mining: an lstm network approach. *IEEE Transactions on Emerging Topics in Computing* **8**, 148–158 (2017).
25. Asim, K., Martínez-Álvarez, F., Basit, A. & Iqbal, T. Earthquake magnitude prediction in hindukush region using machine learning techniques. *Natural Hazards* **85**, 471–486 (2017).
26. DeVries, P. M., Viégas, F., Wattenberg, M. & Meade, B. J. Deep learning of aftershock patterns following large earthquakes. *Nature* **560**, 632–634 (2018).

27. Berhich, A., Belouadha, F.-Z. & Kabbaj, M. I. Lstm-based models for earthquake prediction. In *Proceedings of the 3rd International Conference on Networking, Information Systems & Security*, 1–7 (2020).

28. Al Banna, M. H. *et al.* Attention-based bi-directional long-short term memory network for earthquake prediction. *IEEE Access* **9**, 56589–56603 (2021).

29. Kavianpour, P., Kavianpour, M., Jahani, E. & Ramezani, A. A cnn-bilstm model with attention mechanism for earthquake prediction. *arXiv preprint arXiv:2112.13444* (2021).

30. Sun, J., Xiao, K., Liu, C., Zhou, W. & Xiong, H. Exploiting intra-day patterns for market shock prediction: A machine learning approach. *Expert Systems with Applications* **127**, 272–281 (2019).

31. Zhao, X., Xu, T., Fu, Y., Chen, E. & Guo, H. Incorporating spatio-temporal smoothness for air quality inference. In *2017 IEEE International Conference on Data Mining (ICDM)*, 1177–1182 (IEEE, 2017).

32. Zhang, L. *et al.* Large-scale talent flow forecast with dynamic latent factor model? In *The World Wide Web Conference*, 2312–2322 (ACM, 2019).

33. Abiodun, O. I. *et al.* State-of-the-art in artificial neural network applications: A survey. *Heliyon* **4**, e00938 (2018).

34. Lipton, Z. C., Berkowitz, J. & Elkan, C. A critical review of recurrent neural networks for sequence learning. *arXiv preprint arXiv:1506.00019* (2015).

35. Hochreiter, S. & Schmidhuber, J. Long short-term memory. *Neural computation* **9**, 1735–1780 (1997).

36. Albawi, S., Mohammed, T. A. & Al-Zawi, S. Understanding of a convolutional neural network. In *2017 international conference on engineering and technology (ICET)*, 1–6 (Ieee, 2017).

37. Li, Z., Liu, F., Yang, W., Peng, S. & Zhou, J. A survey of convolutional neural networks: analysis, applications, and prospects. *IEEE transactions on neural networks and learning systems* (2021).

38. Szegedy, C., Vanhoucke, V., Ioffe, S., Shlens, J. & Wojna, Z. Rethinking the inception architecture for computer vision. In *Proceedings of the IEEE conference on computer vision and pattern recognition*, 2818–2826 (2016).

39. Szegedy, C., Ioffe, S., Vanhoucke, V. & Alemi, A. A. Inception-v4, inception-resnet and the impact of residual connections on learning. In *Thirty-first AAAI conference on artificial intelligence* (2017).

40. Vaswani, A. *et al.* Attention is all you need. In *NeurIPS 2017*, 5998–6008 (2017).

41. Devlin, J., Chang, M.-W., Lee, K. & Toutanova, K. Bert: Pre-training of deep bidirectional transformers for language understanding. *arXiv preprint arXiv:1810.04805* (2018).

42. Dai, Z. *et al.* Transformer-xl: Attentive language models beyond a fixed-length context. *arXiv preprint arXiv:1901.02860* (2019).

43. Dosovitskiy, A. *et al.* An image is worth 16x16 words: Transformers for image recognition at scale. *arXiv preprint arXiv:2010.11929* (2020).

44. Liu, Z. *et al.* Swin transformer: Hierarchical vision transformer using shifted windows. In *Proceedings of the IEEE/CVF International Conference on Computer Vision*, 10012–10022 (2021).

45. Song, H., Rajan, D., Thiagarajan, J. J. & Spanias, A. Attend and diagnose: Clinical time series analysis using attention models. In *AAAI 2018* (2018).

46. Zhou, H. *et al.* Informer: Beyond efficient transformer for long sequence time-series forecasting. In *Proceedings of the AAAI Conference on Artificial Intelligence*, vol. 35, 11106–11115 (2021).

47. Radford, A. *et al.* Learning transferable visual models from natural language supervision. In *International Conference on Machine Learning*, 8748–8763 (PMLR, 2021).

48. Kim, W., Son, B. & Kim, I. Vilt: Vision-and-language transformer without convolution or region supervision. In *International Conference on Machine Learning*, 5583–5594 (PMLR, 2021).

49. Wessel, P. *et al.* The generic mapping tools version 6. *Geochemistry, Geophysics, Geosystems* **20**, 5556–5564 (2019). URL https://onlinelibrary.wiley.com/doi/abs/10.1029/2019GC008515. _eprint: https://onlinelibrary.wiley.com/doi/pdf/10.1029/2019GC008515.
